# Allele-specific RNA-seq expression profiling of imprinted genes in mouse isogenic pluripotent states

**DOI:** 10.1186/s13072-019-0259-8

**Published:** 2019-02-15

**Authors:** René A. M. Dirks, Guido van Mierlo, Hindrik H. D. Kerstens, Andreia S. Bernardo, Julianna Kobolák, István Bock, Julien Maruotti, Roger A. Pedersen, András Dinnyés, Martijn A. Huynen, Alice Jouneau, Hendrik Marks

**Affiliations:** 1 Department of Molecular Biology, Faculty of Science, Radboud Institute for Molecular Life Sciences (RIMLS), Radboud University, 6500 HB Nijmegen, The Netherlands; 2 Present Address: Department of Molecular Biology, Faculty of Science, Radboud Institute for Molecular Life Sciences, Oncode Institute, Radboud University Nijmegen, 6525 GA Nijmegen, The Netherlands; 3 The Anne McLaren Laboratory for Regenerative Medicine, Wellcome Trust- Medical Research Council Cambridge Stem Cell Institute, University of Cambridge, Cambridge, CB2 0SZ UK; 4 Mill Hill Laboratory, The Ridgeway, The Francis Crick Institute, London, NW7 1AA UK; 5 BioTalentum Ltd., Gödöllő, Hungary; 6 UMR BDR, INRA, ENVA, Université Paris Saclay, 78350 Jouy en Josas, France; 7 Present Address: Phenocell SAS, Evry, France; 8 Molecular Animal Biotechnology Laboratory, Szent István University, Gödöllő, Hungary; 9 Centre for Molecular and Biomolecular Informatics (CMBI), Radboud Institute for Molecular Life Sciences (RIMLS), Radboud University Medical Centre, 6525 GA Nijmegen, The Netherlands

**Keywords:** Genomic imprinting, Allele-specific RNA-seq, Mouse embryo, Embryonic stem cells, ESCs, EpiSCs, Pluripotency, Parthenogenetic activation (PGA), Nuclear transfer (NT), Genotyping

## Abstract

**Background:**

Genomic imprinting, resulting in parent-of-origin specific gene expression, plays a critical role in mammalian development. Here, we apply allele-specific RNA-seq on isogenic B6D2F1 mice to assay imprinted genes in tissues from early embryonic tissues between E3.5 and E7.25 and in pluripotent cell lines to evaluate maintenance of imprinted gene expression. For the cell lines, we include embryonic stem cells (ESCs) and epiblast stem cells (EpiSCs) derived from fertilized embryos and from embryos obtained after nuclear transfer (NT) or parthenogenetic activation (PGA).

**Results:**

As homozygous genomic regions of PGA-derived cells are not compatible with allele-specific RNA-seq, we developed an RNA-seq-based genotyping strategy allowing identification of informative heterozygous regions. Global analysis shows that proper imprinted gene expression as observed in embryonic tissues is largely lost in the ESC lines included in this study, which mainly consisted of female ESCs. Differentiation of ESC lines to embryoid bodies or NPCs does not restore monoallelic expression of imprinted genes, neither did reprogramming of the serum-cultured ESCs to the pluripotent ground state by the use of 2 kinase inhibitors. Fertilized EpiSC and EpiSC-NT lines largely maintain imprinted gene expression, as did EpiSC-PGA lines that show known paternally expressed genes being silent and known maternally expressed genes consistently showing doubled expression. Notably, two EpiSC-NT lines show aberrant silencing of Rian and Meg3, two critically imprinted genes in mouse iPSCs. With respect to female EpiSC, most of the lines displayed completely skewed *X* inactivation suggesting a (near) clonal origin.

**Conclusions:**

Altogether, our analysis provides a comprehensive overview of imprinted gene expression in pluripotency and provides a benchmark to allow identification of cell lines that faithfully maintain imprinted gene expression and therefore retain full developmental potential.

**Electronic supplementary material:**

The online version of this article (10.1186/s13072-019-0259-8) contains supplementary material, which is available to authorized users.

## Background

Embryonic stem cells (ESCs) and epiblast stem cells (EpiSCs) are pluripotent cells derived from mouse embryos at embryonic day (E) E3.5–E4.5 and E6–E7, respectively [1–4]. Both ESCs and EpiSCs can be directed to differentiate into a wide variety of mature cell types. Therefore, these cells are important models for pre- and post-implantation embryonic development. As mouse ESCs are highly amenable to genetic manipulation and are capable of colonizing the germline of chimaeric mice [5], they are widely used for the generation of transgenic mice.

ESCs and EpiSCs are also promising tools in regenerative medicine due to their self-renewal and differentiation capacity. To avoid an allogeneic immune response during transplantation of these cells, a matching genotype between donor and recipient cells is of key importance. Induced pluripotency has emerged as one of the main methodologies to derive patient-specific pluripotent cells (iPSCs) by reprogramming of adult stem cells using defined reprogramming factors [6]. Two more traditional approaches to obtain genetically matched pluripotent cells include somatic cell nuclear transfer (NT) and parthenogenetic activation (PGA) [7, 8]. During NT, the nucleus of a donor cell is introduced into an enucleated oocyte, after which ESC-NTs or EpiSCs-NTs are derived from the developing embryo. NT is a relatively inefficient process, but it has a significant advantage over transcription factor-mediated reprogramming in that ESC-NTs largely lack residual parental DNA methylation patterns as observed for iPSCs [9]. PGA involves the stimulation of oocytes to produce diploid ESC-PGAs in the absence of fertilization [8]. Although the genotype of the ESC-PGA cells is different from the female oocyte donor due to meiotic recombination occurring in the oocyte, subsequent matching of the ESC-PGAs based on the major histocompatibility complex antigens allows for engraftment of these cells in mouse recipients [10].

Genomic imprinting is an epigenetic process resulting in the expression of genes in a parent-of-origin specific manner. Current estimates suggest the presence of around 200 imprinted genes in mice, most of them having a role in development and social cognition [11, 12]. The importance of imprinting during embryonic development is further underpinned by the fact that androgenetic mice (derived from two paternal pronuclei) and gynogenetic mice (derived from two maternal pronuclei) are embryonic lethal [13, 14]. This is associated with the imprinted H19 and Dlk1-Dio3 loci, as manipulation of these loci enables the generation of viable and fertile bi-maternal mice [15]. In line with this, proper imprinting of the Dlk1-Dio3 locus in iPSCs is essential for significant contribution to chimaeras and for the derivation of viable all-iPSC mice [16]. Together, these studies show that maintenance of imprinted genes in embryonic cells is essential to retain full developmental potential. Additionally, loss of imprinting is highly correlated with transformation and cancer [17, 18], further highlighting the importance of assaying imprinted loci of cells.

Considering the critical role of imprinting in development [19], imprinted genes in ESC and EpiSC lines have been assayed in previous studies. This showed that mouse ESCs tend to lose imprinting upon prolonged culturing resulting in only few genes showing imprinted expression [20–23], while EpiSCs retain their imprints [24]. In contrast to EpiSCs from normal fertilized mouse embryos, EpiSCs derived from NT were reported to show aberrant expression of imprinted genes [25]. With regard to parthenogenesis, ESC-PGA cells were reported to show a surprisingly high efficiency in their contribution to chimaeric mice. This has been attributed to a loss of aberrant imprinting in the ESC-PGA cells [26], but this analysis was based on a very small number of loci. Interestingly, in human loss of imprinting in conventional ESCs is relatively rare, but occurs much more frequently in human ESC-NTs and iPSCs [27]. Most of the studies assaying imprinted genes were performed on a restricted panel of imprinted genes, or in a non-allele-specific manner (i.e., without the knowledge if a gene is expressed from the maternally or paternally derived allele). There is only few studies that include profiling of imprinting in embryonic tissues, which is important for comparative analysis of ESC lines or EpiSC lines with their founder cells. Therefore, a global overview of allele-specific expression of imprinted genes, and how this contributes to the functionality of the ESCs and EpiSCs, is currently lacking.

To study the degree to which imprinted gene expression is preserved in pluripotent cell lines as compared to their founder mouse embryonic cells in vivo, we performed high-resolution allele-specific RNA-seq on embryonic tissues as well as on ESCs and EpiSCs derived after normal fertilization, NT and PGA. For the embryonic tissues, we focused on the very early embryonic stages (between E3.5 and E7.25 [28]) from which ESCs and EpiSCs are derived. Although two recent studies performed in-depth analysis of allelic gene expression during embryonic development, these studies focused on much later stages (from E9.5 onward) [11, 20]. Furthermore, previous studies were carried out on a variety of mouse genetic backgrounds, which is known to influence the efficiency of ESC derivation and to affect NT and PGA [29–32]. In our study, we make use of isogenic F1 hybrids of C57BL/6J (maternal) and DBA/2J (paternal) mouse inbred strains, previously also referred to as B6D2F1, B6D2 or BDF1. This cross has been shown to be relatively efficient in NT and PGA, and the use of isogenic tissues and cell lines eliminates complications arising from strain-specific differences in gene expression [30, 31, 33]. Importantly, when derived from hybrid F1 mice, ESC-PGAs contain a mosaic homo- and heterozygous genotype due to chromosomal crossover occurring in the parental B6D2F1 oocyte during meiosis [10]. Since only the heterozygous part of the genome is compatible with allele-specific expression in RNA-seq, we developed a robust algorithm to reconstruct genotypes of cells derived by PGA based on the RNA-seq profiles we generated. This allowed us to gain insight in allele-specific gene expression of PGA cells at a global scale for the first time.

## Results

### Experimental setup to study imprinted gene expression using allele-specific RNA-seq

**Fig. 1:**
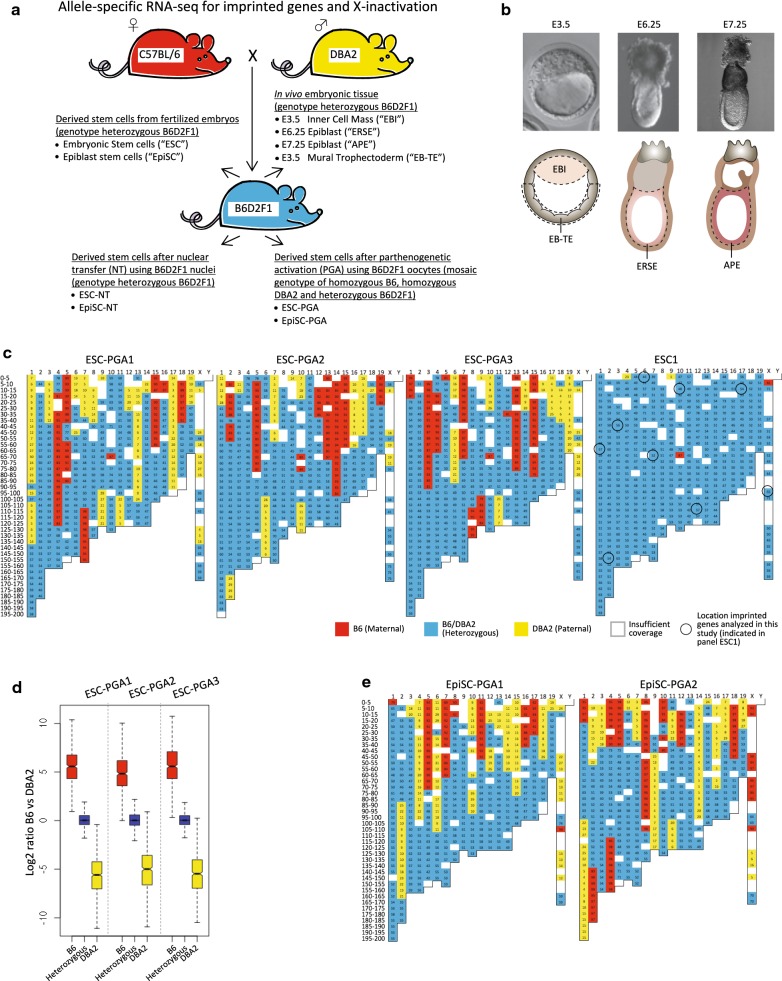
Setup of our study including reconstruction of PGA genotypes using allele-specific RNA-seq. **a** Mouse strains and crosses used to derive the B6D2F1 samples as profiled by RNA-seq. **b** Embryos were collected at different stages of development (top) and microdissected for RNA-seq as shown in the illustration (bottom) [28]. The epiblast is depicted in shades of pink, trophectoderm is in gray, extraembryonic endoderm is light brown, and extraembryonic mesoderm is dark brown. Dashed lines show the cells dissected for total RNA isolation. **c** Genotype of ESC lines as determined by allele-specific RNA-seq at 5 MB resolution. The horizontal axis represents chromosomes, and the vertical axis chromosomal bins (per 5 MB). The numbers within each bin (also categorized by the three colors) represent the percentage B6 as compared to the total coverage of B6 and DBA2. The panel of ESC1 shows the position of imprinted gene clusters that are included in follow-up analysis (Fig. 2). Additional file 2: Fig. S3 shows the genotype of all 8 B6D2F1 ESC lines profiled. **d** Distribution of relative expression of the B6 versus the DBA2 allele of the genes present within genomic regions genotyped as either homozygous B6 (red), heterozygous B6/DBA2 (blue) or homozygous DBA2 (yellow) in the ESC-PGAs. A log2 ratio of 0 represents equal biallelic gene expression from the B6 and DBA2 alleles, while positive and negative ratios represent higher expression from the B6 or DBA2 allele, respectively. **e** Similar to panel **c**, but showing EpiSC lines. Additional file 2: Fig. S5 shows the RNA-seq-based genotyping of all 8 DBA2 EpiSC lines profiled

To assay imprinted genes during embryogenesis and in the ESCs and EpiSCs, we made use of the B6D2F1 mouse model (Fig. 1a). Being an intercross of the genetically distant inbred strains C57BL/6J (referred to in this manuscript as B6) and DBA/2J (referred to as DBA2), the B6D2F1 mice contain around 5.2 million single nucleotide polymorphic sites (SNPs) genome-wide, corresponding to ~ 1 SNP per 520 bp [34–37]. These sites can be used to discriminate between expression from either the B6 maternally or the DBA2 paternally derived alleles in B6D2F1 cells by RNA-seq profiling.

To gain insight into the expression of imprinted genes in the developing B6D2F1 embryo in vivo, we used RNA-seq that we generated previously [28] on three replicate tissues of (1) Expanded Blastocyst: Inner cell mass (E3.5; “EBI”); (2) Epithelial cells of the pre-gastrulation Radially Symmetrical Epiblast (E6.25; “ERSE”); and (3) mid-gastrulation Anterior–Posterior Epiblast (E7.25; “APE”). Given the importance of imprinting in the placenta [38], Expanded Blastocyst: mural TrophEctoderm tissue (E3.5; “EB-TE”; two replicates), we performed RNA-seq profiling to provide insight into imprinted gene expression in the very early extraembryonic trophoblast lineage (Fig. 1a, b; Additional file 1: Table S1). With regard to the pluripotent cell lines, we performed RNA-seq on conventional B6D2F1 ESCs derived from fertilized embryos (two lines, referred to as ESC or fertilized ESC). We also included three ESC lines derived from nuclear transfer embryos generated using B6D2F1 mouse embryonic fibroblast donor nuclei (ESC-NT1) or B6D2F1 donor nuclei of cumulus cells (ESC-NT2 and ESC-NT3), as well as three ESC lines derived from PGA embryos generated using B6D2F1 oocytes (ESC-PGA) (Fig. 1a; Additional file 1: Table S1). Similarly, three replicas of fertilized B6D2F1 EpiSC lines and EpiSC lines derived from nuclear transfer embryos generated using a B6D2F1 donor nuclei of cumulus cells (EpiSC-NT) were included for RNA-seq (Fig. 1a; Additional file 1: Table S1). Lastly, we included two EpiSC-PGA lines which were previously described as EpiSC-NT lines [25], but which we here confirm to be of PGA origin (as further outlined below). As prolonged culture of ESCs has recently been associated with erosion of genomic imprints [39, 40], we used early passage ESCs (between passage 8 and 14; Additional file 1: Table S1). Using regular (non-allele specific) expression analysis of the RNA-seq, we validated the developmental stages of the samples and the RNA-seq profiling (Additional file 2: Fig. S1 and Fig. S2).

### Genotyping of PGA samples using RNA-seq profiling

Cells containing a B6D2F1 genotype, such as the embryonic tissues and most of the cell lines included in this study, are compatible with global allele-specific analysis by RNA-seq. However, only part of the genome of B6D2F1-derived ESC-PGA lines can be utilized to call allele-specific expression using RNA-seq. During the most widely used method for PGA (resulting in p(MII)ESCs referred here as ESC-PGAs), metaphase II-halted oocytes are stimulated to proceed through meiosis in the absence of fertilization. Blocking the extrusion of the second polar body results in a diploid parthenote. Subsequent chemical activation toward further development allows for derivation of ESC-PGAs [8]. Metaphase II oocytes have already undergone meiotic recombination, and therefore, the genotype of the subsequent ESC-PGA cells is different from the female oocyte donor: B6D2F1 ESC-PGA lines are homozygous proximal to the centromere, while they are heterozygous at distal regions for most chromosomes [10]. The homozygous parts of the genome of the PGA lines lack SNPs required for allele-specific RNA-seq analysis. Thus, only the heterozygous parts of the genome of PGA lines can be utilized to call allele-specific expression. To identify these heterozygous parts, we used the allele-specific RNA-seq mapping counts over the B6/DBA2 polymorphic sites to calculate the relative contribution of either B6 (maternal) or DBA2 (paternal) in genomic bins, on which we applied a moving average over the individual chromosomes. Since we expect only 1 or few breakpoints per chromosome, we first analyzed at a low resolution to gain a comprehensive overview. Using a low-resolution binning size of 5 Mb (Fig. 1c), this RNA-seq SNP genotyping very clearly shows a pattern of recombination in all the ESC-PGAs. In contrast, the fertilized ESCs and ESC-NTs show a full heterozygous F1 genotype as expected (Fig. 1c, Additional file 2: Fig. S3). To better estimate the chromosomal breakpoints, we increased our resolution to 1 MB (Additional file 3: Table S2). Plotting of the allelic ratios of genes present within the parts of the genome in ESC-PGAs annotated as either B6, heterozygous or DBA2 shows the expected pattern (Fig. 1d). Genes present in the heterozygous part of the genome are largely expressed from both alleles, while alleles of genes present in the homozygous part of the genome cannot be discriminated. Therefore, these genes show a (near) complete bias according to their genotype. Together, this allowed us to only include genes present in the informative heterozygous parts in the genome (Additional file 3: Table S2) for further allele-specific RNA-seq analysis of the PGA lines.

To verify the genotype of the remaining embryonic tissues and EpiSC samples, we applied the same RNA-seq-based genotyping strategy. The in vivo embryonic tissues showed the expected heterozygous F1 genotype, except for the maternal B6 bias on the *X* chromosome which reflects the presence of male embryos in the pooled male and female embryos samples (Additional file 2: Fig. S4). All fertilized EpiSC and three of the EpiSC-NT lines also showed the expected F1 genotype as expected (Additional file 2: Fig. S5). Additionally, we identified two parthenogenetic EpiSC lines (Fig. 1e). These EpiSC-PGA lines have originally been reported as being derived after transfer of B6D2F1 donor nuclei of cumulus cells [25]. Nevertheless, they show a clear mosaic genotype, not matching the expected F1 genotype as observed for the other three EpiSC-NT lines (Additional file 2: Fig. S5). Instead, these two EpiSC lines show a recombination pattern unique to PGA (Fig. 1e; cf Fig. 1c). To verify these two unexpected genotypes, we performed regular genotyping using genomic DNA, yielding results nearly identical to the RNA-seq genotyping, both at 5 MB resolution (Additional file 2: Fig. S6; cf Additional file 2: Fig. S5) and at 1 MB resolution (Additional file 4: Table S3). This further confirms the mosaic PGA-specific genotypes for both EpiSC lines, and these lines were included in this study accordingly. The accidental isolation of PGA-derived cells during nuclear transfer experiments is a serious concern [41] and is likely due to improper enucleation preceding the nuclear transfer resulting in PGA of the original nucleus after stimulation of the oocyte.

Finally, to validate our RNA-seq-based genotyping approach, we made further use of the direct comparison with regular DNA-based genotyping. At 1 MB resolution, the RNA-seq genotyping algorithm identified the exact same position of genomic breakpoints in 56% (23 of 41) of the cases as compared to DNA-based genotyping, while it shows an average accuracy of 2.9 MB in the EpiSC-PGAs (Additional file 4: Table S3). Notably, our RNA-seq-based genotyping algorithm also allows for identification of genomic alterations (Additional file 2: Fig. S8). Together, this shows the accuracy and sensitivity of our RNA-seq-based genotyping approach and further confirms our detection of the heterozygous parts of the PGA cell lines to be used in further analysis for imprinting.

### Allele-specific expression of imprinted genes in embryonic tissues

**Fig. 2:**
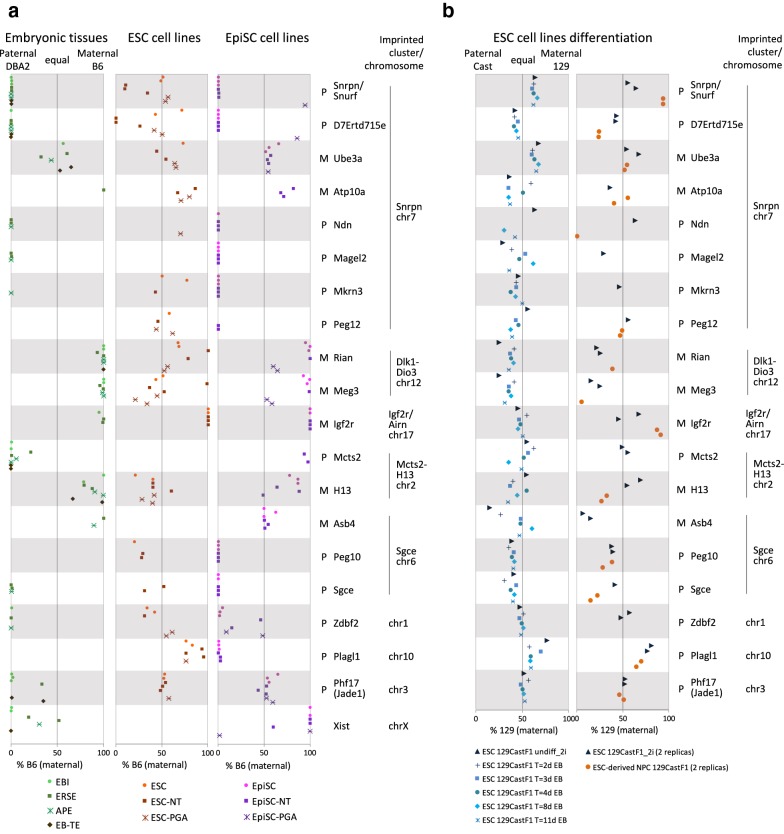
Allele-specific expression of imprinted genes in mouse in vivo and in vitro pluripotent states. **a** Allelic bias in expression of known imprinted genes plotted by the percentage of B6 as compared to the total coverage of B6 and DBA2 per gene. Within individual panels, the left axis represents expression from the DBA2 (paternal) allele, the right axis represents expression from the B6 (maternal) allele, and the middle axis represents equal biallelic expression. The individual replicas of all samples are included in the graph, but not individually labeled. The graph only includes data points of genes for which we obtained sufficient coverage to calculate allelic bias, explaining the variable number of data points between genes or the complete lack of data points for some genes in either of the cell lines or the embryonic tissue. “*P*” = paternally expressed; “*M*” = maternally expressed. **b** Similar to **a**, showing allelic expression of imprinted genes during differentiation of ESCs toward embryoid bodies (left panel) or NPCs (right panel)

For allele-specific expression, we applied a computational pipeline for unbiased allele-specific quantification of transcripts that we developed previously, obtaining quantitative allelic information for up ~ 5000 genes per sample (Additional file 2: Fig. S9) [42]. On average, individual genes show equal expression from the B6- and DBA2-derived alleles in all samples, including in the heterozygous chromosomal regions of the PGA-derived lines, with only a small number of genes showing allelic bias in expression toward B6 or DBA2 as would be expected for imprinted genes (Additional file 2: Fig. S9). To determine allele-specific expression of imprinted genes, we compiled a list of 98 imprinted genes based on available data present in the two most widely used imprinting databases (www.geneimprint.com [43] and www.har.mrc.ac.uk/research/genomic_imprinting/ [44]) and in a recent comprehensive survey on imprinted genes over a large number of developmental stages and tissues [11] (Additional file 5: Table S4). Of these 98 genes, 20 genes had sufficient RNA-seq coverage over polymorphic sites (> 9 counts [42]) to quantify allele-specific expression in at least one of the samples included in our analysis (Fig. 2a; Additional file 6: Table S5). Notably, in order to obtain reliable allelic ratios, we only included a gene of an individual sample in our analysis if it showed consistent B6 versus DBA2 allelic ratios over all polymorphic sites that are present within the gene body (standard error of the mean < 0.15; see Methods section for more details). An overview of the RNA-seq data of all B6D2F1 samples over a selection of the imprinted genes is provided in Additional file 2: Fig. S10.

For the in vivo embryonic tissues, we obtained allelic information for 16 autosomal genes and 1 *X*-linked gene (Xist), while expression of Peg12, Peg10 and Plagl1 was too low in any of the samples to reliably call allelic ratios (Fig. 2a). From the 16 autosomal imprinted genes covered, 13 (81%) follow a pattern of (nearly) full imprinting in all samples for which we obtained allelic ratios (Fig. 2a). Another two genes (13%) show a strong parental bias (> 80%) toward either the maternal (H13) or the paternal allele (Phf17), in line with later embryonic stages or tissues for which these genes consistently show an allelic bias but not complete monoallelic expression [11, 45]. Interestingly, the imprinting pattern of the EB-TE trophectoderm samples was nearly identical to that observed for the other embryonic tissues, including the EBI embryoblast tissues obtained from the same embryonic stage E3.5. Previous studies focusing on mouse embryonic tissues at later stages (E9.5 and later) showed that imprinting of Phf17 was restricted to the mouse placenta and yolk salk [11]. Here, we find that Phf17 also consistently shows allelic expression bias in early mouse embryonic inner cell mass tissue. This suggests that imprinting of Phf17 outside extra-embryonic tissue is lost shortly after the blastocyst stage. Ube3a, the only gene that consistently shows biallelic expression at all embryonic stages profiled, was originally identified as being imprinted in brain [46]. Apparently, this gene is not imprinted during embryonic development. Finally, it should be noted that the individual in vivo embryonic tissues originate from a pool of male and female embryos, which complicates interpretation of allelic ratios of the *X*-linked gene Xist for these samples. Together, these results show that we robustly detect imprinting in B6D2F1 early (extra-)embryonic tissue for nearly all known imprinted genes covered in our transcriptomes, extending previous observations of highly conserved imprinting pattern across tissues [11] to early embryonic and extra-embryonic tissues.

### Allele-specific expression of imprinted genes in ESCs

Most of the ESC lines, either fertilized ESCs, ESC-NTs or ESC-PGAs, show a very different pattern of imprinted gene expression as compared to the in vivo embryonic tissues, including the E3.5 inner cell mass tissue that they originate from (Fig. 2a). In the fertilized ESCs, 11 out of the 13 imprinted genes that contain sufficient coverage show biallelic expression in both ESC lines (Fig. 2a; Additional file 6: Table S5). Considering the pattern observed in the embryonic tissues, this strongly suggests that imprinted gene expression in fertilized ESCs is lost during outgrowth or maintenance of ESCs. In line, two out of three ESC-NT lines (ESC-NT2 and ESC-NT3) and all three ESC-PGA lines show biallelic expression for the majority of imprinted genes that contain sufficient coverage (11 out of 16 autosomal genes). In contrast to a previous study [47], we find monoallelic (imprinted) expression of Igf2r in the ESCs. This difference in allelic bias of Igf2r might be caused by a difference in mouse strains used, as we also find monoallelic expression of Igf2r in the B6D2F1 embryonic tissues (Fig. 2a). To gain further insight into the loss of imprinted gene expression, we performed allele-specific ChIP-Seq profiling of the posttranslational histone modifications H3K4me3 and H3K27me3, epigenetic marks associated with gene activity or gene silencing, respectively [48]. In line with the biallelic expression of imprinted genes in ESCs, the imprinted monoallelic presence of H3K4me3 and H3K27me3 is largely lost in B6D2F1 fertilized ESCs, ESC-NTs or ESC-PGAs (Additional file 2: Fig. S11 and Fig. S12). Interestingly, one NT-derived ESC line (ESC-NT1) showed an allelic bias of > 80% for most of the genes which were covered by the allele-specific RNA-seq (Snrpn/Snurf, D7Ertd715e, Rian, Meg3 and Igf2r), similar to the monoallelic expression observed in the embryonic tissues (Fig. 2a). This suggests that the loss of monoallelic expression of imprinted genes observed for ESC-NT2 and ESC-NT3 is likely associated with the derivation and maintenance of the ESCs rather than with the nuclear transfer per se. Furthermore, this shows that occasionally ESC lines (largely) retain imprinted gene expression, in line with previous observations on the variation of imprinted gene expression in ESCs [24].

To investigate whether the observed loss of imprinted gene expression was B6D2F1-strain specific, we analyzed allele-specific RNA-seq profiles from fertilized ESCs originating from a cross between mouse *strains Mus musculus (M.m.) domesticus* 129/SV-Jae (129) and *M.m. castaneus* (Cast) (129xCast) that we generated previously (Additional file 2: Fig. S13; ESC 129CastF1 [42]). Similar to the B6D2F1 ESCs, almost all imprinted genes were biallelically expressed [< 80% allelic bias for 12 out of 13 genes (92%)], showing that the observed loss of imprinting in ESCs is independent of the genotype. We next asked whether the loss of imprinted gene expression is dependent on culture conditions. The B6D2F1 and 129CastF1 ESC lines are maintained in the presence of serum [either supplemented with LIF (129CastF1 ESCs) or on feeder cells (B6D2F1 ESCs)]. It has been shown that culturing ESCs in serum-free minimal media containing 2 kinase inhibitors (“2i”) maintains or reverts ESCs toward the ground state of pluripotency, resulting in an ESC transcriptome resembling the early in vivo tissue of the inner cell mass [49–52]. We therefore adapted the 129CastF1 ESCs to 2i media (> 12 days) and assayed imprinted genes by allele-specific RNA-seq. Adaptation to 2i did not result in major changes in the ratio of allelic expression of imprinted genes, and the majority of imprinted genes (17/19; 89%) still showed biallelic expression (Additional file 2: Fig. S13; ESC 129CastF1_2i). These results show the strong tendency of ESCs to lose imprinted gene expression.

The observed biallelic expression of imprinted genes in ESCs is likely to be the consequence of (1) a loss of epigenetic imprints at the imprinted control regions (ICRs); and/or (2) a relaxation in the ESCs to “read” these epigenetic imprints. Also, for some imprinted genes monoallelic expression only arises during development [53]. To further investigate these issues, we differentiated ESCs to embryoid bodies (EBs) or neural progenitor cells (NPCs). EB differentiation of either 2i ESCs or serum ESCs did not change the biallelic expression of imprinted genes as observed in the undifferentiated ESCs (Fig. 2b; Additional file 2: Fig. S14). Most of the imprinted genes also remained biallelically expressed in the NPCs generated from the ESCs (Fig. 2b). Together, this shows that ESCs tend to lose imprinting and that imprints are not reinstalled upon differentiation of the ESCs toward developmentally more advanced cell types.

### Allele-specific expression of imprinted genes in EpiSCs

**Fig. 3:**
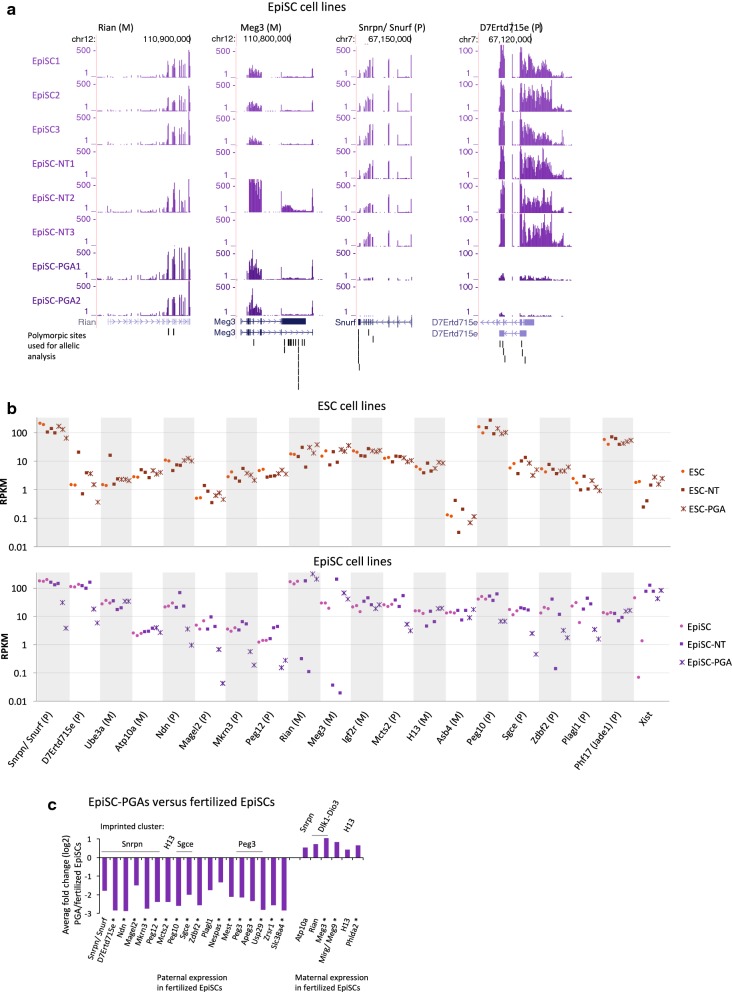
Misexpression of imprinted genes in EpiSC-NTs and EpiSC-PGAs. **a** Example of tag-normalized RNA-seq data over imprinted genes in EpiSCs. A further extension of this example is shown in Additional file 2: Fig. S10. **b** Quantification of expression levels (RPKM) of the genes shown in Fig. 2a. The individual replicas of all samples are included in the graph, but not individually labeled. “*P*” = paternally expressed; “*M*” = maternally expressed. Additional file 2: Fig. S15 additionally includes quantification of expression levels of the B6D2F1 embryonic tissues. **c** Average fold change in gene expression in EpiSC-PGAs as compared to fertilized EpiSCs. Statistically significant changes (adjusted *p* value < 0.05) are indicated with a asterisk. A detailed genome browser view for Snrpn/Snurf and D7Ertd715e is shown in **a**. Besides imprinted genes included in Fig. 2a, this panel additionally includes all other known imprinted genes differentially expressed between EpiSCs and EpiSC-PGAs (Additional file 7: Table S6)

In contrast to ESCs, fertilized EpiSCs and EpiSC-NTs largely retain allele-specific expression of imprinted genes as compared with the in vivo embryonic tissues, including their in vivo counterparts ERSE and APE (Fig. 2a). A total of 10 of the 15 genes that were imprinted or biased in the embryonic tissues show full imprinting in EpiSC and EpiSC-NT lines, while another two genes (Atp10a and H13) show a strong allelic bias. Additionally, Peg12, Peg10 and Plagl1, for which we obtained insufficient coverage of the RNA-seq in the embryonic tissues, show imprinted gene expression in the fertilized EpiSC and EpiSC-NT lines. Mcts2, Asb4 and Phf17 lost imprinted gene expression in the fertilized EpiSCs and EpiSC-NTs as compared to the embryonic tissues (Fig. 2a). Notably, Rian and Meg3 (also known as Gtl2) have very high coverage in the fertilized EpiSCs and in EpiSC-NT2, but lack sufficient coverage of the RNA-seq to determine allelic bias in the EpiSC-NT1 and EpiSC-NT3 (Fig. 2a; Additional file 6: Table S5). In line, expression of Rian and Meg3 is nearly absent in EpiSC-NT1 and EpiSC-NT3, in contrast to the high expression observed in the other EpiSC lines (Fig. 3a, b). This is reminiscent of the aberrant Rian and Meg3 silencing in iPSC lines, for which proper imprinting of both genes is essential to retain full developmental potential [16].

To study the effect of nuclear transfer at the total expression level of imprinted genes, we performed regular (non-allele specific) gene expression analysis of fertilized EpiSCs and EpiSC-NTs. Although only few imprinted genes were present among all differentially expressed genes (adjusted *p* value < 0.05; Additional file 7: Table S6), the imprinted genes Igf2 and Cdkn1c (p57) were among the most significant (Additional file 2: Fig. S16; Additional file 7: Table S6). Imprinting of Igf2 is relatively unstable [54] and loss of imprinting might therefore partly underlie the increased expression of Igf2 in the EpiSC-NT lines. However, Igf2 as well as Cdkn1c do not contain SNPs in the B6D2F1 genotype to confirm loss of imprinting in the current study. Functionally, Igf2 and Cdkn1c are important in control of cell proliferation and development [55]. As such, misregulation of these genes as observed in EpiSC-NT cells might provide a selective growth advantage, as previously suggested for Igf2 in human ESCs [54].

Calling of allele-specific bias on the B6D2F1 heterozygous part of the EpiSC-PGA genomes clearly shows a (near) complete loss of monoallelic expression of imprinted genes, in line with the absence of paternal imprinting (Fig. 2a). For known paternally expressed genes included in Fig. 2a, this resulted in an average drop in expression of > fourfold in the EpiSC-PGAs (Fig. 3b, c). Seven of the twelve paternal imprinted genes as shown in Fig. 2a show a significant change in expression (adjusted *p* value < 0.05; Additional file 7: Table S6). The same trend was observed for other well-known paternally imprinted genes for which the B6D2F1 genotype does not have allelic coverage, such as Peg3 (Fig. 3c). In line, of all 77 genes that are significantly downregulated in EpiSC-PGAs as compared to fertilized EpiSCs (adjusted *p* value < 0.05), the top most downregulated genes are known imprinted genes (Additional file 7: Table S6). In contrast to paternally expressed genes, maternally expressed genes generally showed a minor (but occasional significant) upregulation in the EpiSC-PGAs. While no maternally expressed genes were significantly downregulated, three were significantly upregulated (out of a total of 47 upregulated genes (adjusted *p* value < 0.05) between EpiSC-PGAs and fertilized EpiSCs (Additional file 7: Table S6; Fig. 3c). The observed ~ twofold increase is in agreement with the lack of a silent, imprinted paternal allele in the EpiSC-PGAs. To gain further insight in the regulation of imprinted genes in EpiSC-PGAs, we performed global DNA methylation profiling using MethylCap-Seq. Analysis of the ICRs belonging to the imprinted genes in the EpiSC-PGAs showed (1) a loss of DNA methylation on the ICR for paternally imprinted (maternally expressed) genes; and (2) a twofold gain of DNA methylation on the ICR for maternally imprinted (paternally expressed) genes (Additional file 2: Fig. S17). Altogether, these results indicate that both alleles in the EpiSC-PGAs contain a maternal imprinting pattern, and therefore, imprinting in the EpiSC-PGAs is correctly maintained as both alleles are maternally derived. Interestingly, these observations in EpiSC-PGAs are different from ESC-PGAs, which tend to show a loss of genomic DNA methylation imprints (Fig. 2a) [26].

### X inactivation in EpiSC: EpiSC clonality

For most of the EpiSC lines, we noticed a full allelic bias in Xist expression associated with *X* inactivation (Fig. 2a). Random *X* inactivation (rXCI) occurs in the mouse between E5.5 and E7.5 during which individual female cells in the epiblast randomly inactivate one of the two *X* chromosomes, which is stably propagated to daughter cells [56]. Consequently, female founder cells of EpiSCs have usually undergone rXCI and EpiSC lines contain an active (Xa) as well as an inactive *X* chromosome (Xi) [57]. The noncoding RNA Xist is required for *X* inactivation and is specifically expressed from the Xi [58, 59]. Considering that B6- and DBA2-derived *X* chromosomes have an equal chance to be inactivated during rXCI [as both contain the same *X*-controlling element “b” locus (Xce^b^)] [60], the ratio of female B6D2F1 cells within the epiblast that contain either an inactivated B6- or DBA2-derived *X* chromosome is around 0.5. Similarly, in case (some) founder cells initiate rXCI during derivation or early culturing of the EpiSC lines, the ratio is also around 0.5. Therefore, we expected that half of the cells within female EpiSC lines would contain an inactive B6-derived *X* chromosome, while the other half of the cells would contain an inactive DBA2-derived one. At population level of stable EpiSC lines, this would be represented by an equal allelic expression of Xist from the B6- and DBA2-derived *X* chromosome. However, most of the EpiSC lines that we included in our analysis show a full allelic bias of Xist (Fig. 2a).

**Fig. 4:**
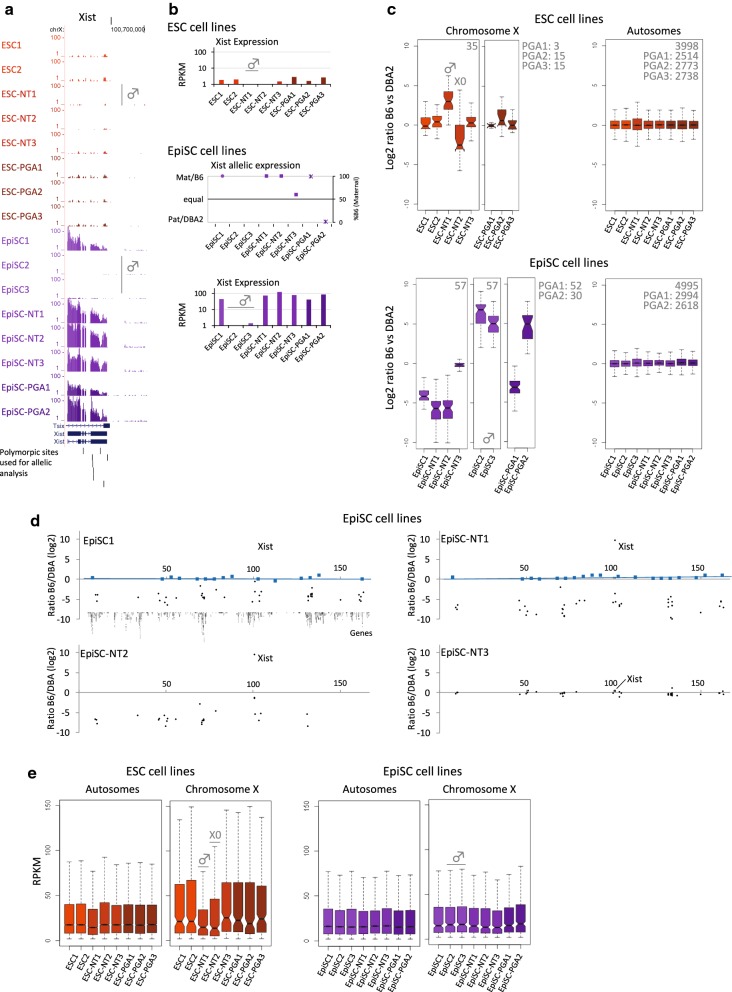
Completely skewed *X* inactivation in B6D2F1 EpiSCs. **a** Tag-normalized RNA-seq data over Xist gene. **b** Quantification of Xist expression (RPKM), including allelic bias for the EpiSCs. Expression of Xist in the ESCs is too low to quantify allelic bias. **c** Distribution of relative expression of genes from the B6 versus the DBA2 allele in the ESC and EpiSC lines over chromosome *X* or autosomes (control). A log2 ratio of 0 represents equal gene expression from the B6 and DBA2 alleles, while positive and negative ratios represent higher expression from the B6 or DBA2 allele, respectively. On top the number of genes included in each of the boxplots, for the PGA lines we only included genes in the heterozygous B6/DBA2 parts. Besides ESC-NT1, EpiSC2 and EpiSC3, all lines are female. Note that the EpiSC-PGAs contain a largely heterozygous *X* chromosome including the Xist locus, enabling allele-specific RNA-seq calls (Additional file 4: Table S3). **d** B6/DBA2 ratio per gene over the linear *X* chromosome (the *X*-axis represents genomic coordinates in MB). Each dot represents a gene, and 50 genes had sufficient coverage over SNPs to be included. In blue (for female EpiSC1 and EpiSC-NT1) the B6/DBA2 ratio obtained by DNA sequencing at 5 MB resolution, confirming the presence of a B6 and DBA chromosome *X* in both lines. Additional file 2: Fig. S18 includes the same graphs for all B6D2F1 fertilized and NT ESCs or EpiSCs included in this study. **e** Distribution of gene expression in ESC and EpiSC lines over chromosome *X* or autosomes (control). The lower expression of *X*-linked genes in EpiSCs as compared to ESCs is the consequence of XCI in the EpiSCs resulting in dosage compensation between expression of autosomes and chromosome X. Genes with an expression level of RPKM > 2 in all ESC (246 and 8416 genes on chromosome *X* and autosomes, respectively) or EpiSC lines (332 and 9382 genes on chromosome *X* and autosomes, respectively) are included in this analysis

To further investigate the allelic bias of Xist, we examined expression of Xist and other *X*-linked genes. As expected, Xist is highly expressed in all six female EpiSC lines, but not in the seven female ESC lines as the female ESC founder cells have not yet undergone rXCI. Also, Xist expression is absent in the single male ESC and the two male EpiSC lines that were included in this study (Fig. 4a, b). Independent of their origin (fertilized EpiSC, EpiSC-NT or EpiSC-PGA), five of the six female EpiSC lines showed full bias in allelic expression of Xist, with expression from the B6-derived *X* chromosome being observed in most of the EpiSC lines (Fig. 4b). In line with the allelic bias of Xist in these five EpiSC lines, we observed a (near) monoallelic expression of the far majority of *X*-linked genes reciprocal to the allelic ratio of Xist (Fig. 4c, d). EpiSC-NT3 is the only line which showed equal expression of *X*-linked genes from both B6 and DBA2 alleles (Fig. 4c, d), including Xist (Fig. 4b). We observed biallelic expression and an increased global expression of *X*-linked genes in the female ESC lines (except for ESC-NT2), in keeping with the presence of two active *X* chromosomes in ESCs (Fig. 4c, e; Additional file 2: Fig. S18a). ESC-NT1 is male and, therefore, only shows B6-derived RNA-seq signals from the *X* chromosome (Fig. 4c; Additional file 2: Fig. S18a). As female ESCs are prone to the loss of one *X* chromosome, we analyzed the karyotype of the ESC lines as a control (Additional file 1: Table S1; [61]). This showed that the ESC-NT2 line is largely X0, explaining the allelic bias and the reduction in *X*-linked gene expression for ESC-NT2 (Fig. 4c, e; Additional file 2: Fig. S18a). Together, our results indicate that 5 of the 6 female EpiSC lines show a complete skewing of XCI, with the DBA2-derived *X* chromosome being active (Xa) in 4 EpiSC lines and the B6-derived *X* chromosome being active (Xa) in the EpiSC-PGA2 line. The most likely explanation for the observed skewing is that the EpiSC lines are (largely) of clonal origin, as further outlined in the discussion. Only occasionally, EpiSC lines are derived from a mixed population of cells, as is the case for the EpiSC-NT3 line which consists of a mixed population of cells containing either an inactive B6- or an inactive DBA2-derived *X* chromosome. Since derivation of the EpiSCs was performed using standard protocols [1, 25, 62], clonality is likely to be a common phenomenon among EpiSC lines.

## Discussion

Genomic imprinting is critical for proper development, as alterations in imprinting gene dosage in early embryos are often associated with development defects (reviewed by Peters [63]). In line, we demonstrate that parent-specific monoallelic expression of imprinted genes is intact in pre- and post-implantation embryos. Within in vitro cultures, maintenance of imprinted gene expression in embryonic cells is essential to retain full developmental potential [13–16]. Considering this critical role, previous studies have assayed allelic bias of imprinted gene expression in fertilized ESCs and EpiSCs [24, 54, 64, 65]. However, most of these studies were performed on a restricted panel of imprinted genes by targeted sequencing of expressed SNPs in cDNA. In case of NT and PGA, allelic bias of imprinted genes in mouse ESCs or EpiSCs has mainly been assayed by non-allele-specific DNA methylation profiling, either at a genome-wide scale or using more targeted approaches [25, 26, 66–68]. Information on allele-specific expression of imprinted genes in NT and PGA lines is largely absent. Importantly, most of the studies investigating imprinting in ESCs and EpiSCs did not include analysis of the embryonic tissues and therefore cannot discriminate between lack of imprinting in embryonic tissue versus loss (or gain) of imprinting during derivation of ESCs and/or EpiSCs. The analysis performed here therefore extends previous studies by providing a comprehensive overview of imprinted gene expression in pluripotency. The use of isogenic tissues and cell lines in the current study allows for direct comparisons between pluripotent cells and as such provides a benchmark to allow identification of cell lines that faithfully maintain imprinted gene expression. A notable example showing the power of the dataset we generated represents the known imprinted gene Ube3a, for which the biallelic expression observed in the ESC and EpiSC lines is not due to a loss of imprinting, but due to absence of imprinting in the embryonic tissue of origin. It should be noted that the B6D2F1 hybrid mouse strain which we used for our analysis contains relatively few polymorphic sites (~ 5.2 million) as compared to other hybrid mouse crosses that are often used to study allelic gene expression (e.g., a 129xCast hybrid mouse strain contains ~ 20.8 million polymorphic sites [42]). Therefore, the coverage over imprinted genes to be included in our analysis is relatively low. However, the B6D2F1 genotype has been shown to be superior over other mouse strains in nuclear transfer and PGA (both in efficiency and in the development and quality of the embryos or pluripotent cell lines [31, 33, 69, 70]), which we used as main criteria for selecting the B6D2F1 strain for the current study.

To facilitate global allele-specific expression analysis of PGA-derived cell lines, we performed RNA-seq-based genotyping to determine the positions of genomic crossover. A direct comparison with regular DNA genotyping shows that the algorithm is robust and accurate in detecting chromosomal breakpoints. Despite the fact our RNA-seq genotyping method lacks resolution in “gene deserts” because of the absence of coverage of the RNA-seq, the position of the genomic breakpoints called from the RNA-seq exactly matches the DNA-based genotyping in over half of the cases. Previous studies reporting chromosomal breakpoints in human or mouse PGA p(MII)ESC lines used either (1) DNA genotyping of p(MII)ESCs derived from an F1 mouse cross similar to the DNA genotyping performed here used as a control (Additional file 2: Fig. S6) [10]; (2) the signature of heterozygous and homozygous signals over SNPs as obtained by DNA-based genotyping [41] or by RNA-seq [71]. The latter method takes advantage of the fact that p(MII)ES cells retain pericentromeric homozygosity but show distal regions of heterozygosity due to crossing-over in the oocyte [10, 41] and is particularly powerful if parental genotypes are unknown. However, in the absence of parental genotypes, this method cannot discriminate between homozygotic parts of the genome being derived from either the paternal or the maternal parent of the oocyte donor. By taking advantage of mouse inbred strains and the known genomes of the B6 and DBA2 mouse strains, our method enables accurate characterization of the genotype of the PGA-derived ESC and EpiSC lines. This allowed us to comprehensively show for the first time that twofold upregulation of known maternally expressed genes in PGA-derived cells is caused by the activation of the paternal allele (which lacks the imprint in the PGA lines), as these genes show an equal biallelic expression in our allele-specific analysis. Next to its use for PGA lines, our RNA-seq-based genotyping method is applicable to F2 hybrids and subsequent generations of crossbreeds, as well as backcrosses, of (mouse) inbred strains. As such, genotypic information can conveniently be obtained for studies where gene expression analysis by RNA-seq has been performed.

Our genome-wide study shows that mouse ESCs tend to lose imprinting, while EpiSCs retain their imprints, similar to previous observations on a restricted panel of imprinted genes [24]. In contrast to fertilized EpiSCs, EpiSCs derived after NT were reported to show aberrant expression of a large number of imprinted genes [25]. However, this conclusion was largely based on analyses of EpiSC lines that accidentally originated from parthenogenesis (instead of NT), which indeed show loss of imprinted gene expression (Fig. 2a). Our current analyses, including proper genotyping as control for the nuclear transfer, show that allelic expression of imprinted genes is largely retained in EpiSC-NTs (Figs. 2, 3). Despite the similarity of EpiSC-NT lines to embryonic tissues or fertilized EpiSCs, the Dlk1-Dio3 imprinted gene cluster is notably different in expression. In this cluster, Rian and Meg3 are robustly expressed in the fertilized EpiSC lines and one EpiSC-NT line, while expression is absent in two EpiSC-NT lines. Aberrant silencing of Rian and Meg3 has previously been observed in iPSC lines. In contrast to iPSC lines with normal expression of Rian and Meg3, iPSC lines in which these genes are silenced contribute poorly to chimaeric mice and fail to support the development of entirely iPSC-derived mice [16]. The misexpression in the Dlk1-Dio3 imprinted gene cluster has been associated with transcription factor-mediated reprogramming, as it has not been observed in other methods of reprogramming thus far. Although we only profiled a small sample set, our data suggest that silencing of Rian and Meg3 is a more general phenomenon in reprogramming and can also occur during nuclear transfer. In iPSCs, the presence of ascorbic acid during reprogramming prevents misexpression of the Dlk1-Dio3 cluster [72]. It will be interesting to test whether the presence of ascorbic acid after nuclear transfer can similarly preserve a normal imprinting status at the Dlk1-Dio3 gene cluster in EpiSC-NTs. If so, this might underlie the beneficial effect of ascorbic acid on mouse nuclear transfer as reported recently [73].

EpiSCs are derived during in vitro outgrowth of post-implantation epiblasts on which colonies emerge. In order to obtain pure EpiSCs and get rid of the remainder of the mouse epiblast outgrowth, these EpiSC colonies are picked after a week of epiblast culture to be further expanded toward EpiSC lines [1, 25, 62]. It has been observed that the EpiSCs colonies emerging from the epiblast are generally localized close together [25] (Alice Jouneau, personal communication). The close concentration of EpiSC colonies might suggest a clonal origin of EpiSC lines. rXCI randomly occurs in early post-implantation female embryos (generally before collection of the epiblast for EpiSC derivation) and is stably maintained afterward. Therefore, full skewing of XCI is widely used to assess clonality [74]. The use of mouse F1 hybrid in combination with allele-specific RNA-seq allows for convenient detection of skewing, which otherwise often remains unnoticed. Our analysis shows that five of the six female EpiSC lines as profiled here show a complete skewing of XCI (Fig. 4). Therefore, this strongly suggests that these EpiSC lines are clonal, although we cannot exclude that the EpiSC lines originate from two or more founder cells that inactivated the same copy of the *X* chromosome. In line, the EpiSC colonies emerging from the epiblast during derivation of the EpiSC-NT3 line (which showed biallelic expression of Xist) were spread over a much larger area of the epiblast, explaining the EpiSC-NT3 line not being clonal. Altogether, the skewed XCI observed for the majority of EpiSC lines included in the current study suggests that these lines originate from a single cell of the early epiblast that divided further during development and gave rise to multiple colonies during in vitro outgrowth, which were picked for further expansion. The discrete areas showing outgrowth of EpiSC colonies might reflect regions of the epiblast that are more permissive toward EpiSC derivation than other areas. This could be due to the heterogeneous nature of the epiblast, which contains a range of cells with different commitment toward the various embryonic lineages [75], and of which only a few cells might be susceptible for outgrowth toward EpiSC lines.

Collectively our observations yield insight into global imprinted gene expression patterns during early embryonic development, and the cell lines derived from these early embryonic stages. Thereby, it provides a benchmark to allow identification of cell lines that faithfully maintain imprinted gene expression and therefore retain full developmental potential. Our study provides further evidence that, in contrast to EpiSCs, ESCs are prone to lose allelic expression of imprinted genes. It should be noted that the majority of ES cell lines included in this study were female, which are more susceptible to losing imprinting as compared to male ESCs due to their hypomethylated state [76]. Also, it has been shown that prolonged in vitro culture of ESCs in serum containing media perturbs the maintenance of methylation imprints that control the allelic expression of imprinted genes [20–23], which for the ESC lines included in this study largely occurred within 20–30 days (8–14 passages; Additional file 1: Table S1). EpiSC lines as included in this study were cultured in serum-free medium (CDM [77]), which might facilitate the maintenance of imprinting in EpiSCs, for both male and female EpiSCs (Additional file 1: Table S1). In recent years, the use of kinase inhibitors (“2i”) within a serum-free defined medium has enabled derivation of mouse ground state pluripotent ESCs that more faithfully reflect early in vivo cells of the inner cell mass as compared to conventional ESCs cultures in serum and LIF [49–52, 78]. Despite the fact that adaptation of conventional ESCs to 2i allows for reprogramming of the epigenome to the ground state [50, 79, 80], it does not rescue the imprinting pattern of founder embryonic cells (Additional file 2: Fig. S13). This is in line with very recent studies showing that the use of 2i-conditions results in erosion of DNA methylation at most ICRs in ESCs [39, 40]. Importantly, we show that the allelic expression of imprinted genes is not restored upon differentiation of the ESCs, while also conversion of ESCs to EpiSCs does not restore imprinting patterns [24]. Together, this strongly suggests that the loss of imprinted gene expression as observed in ESCs is caused by an irreversible loss of epigenetic imprints (i.e., a loss of DNA methylation (or other epigenetic marks [81]) at ICRs), rather than a relaxation in ESCs to translate these imprints to monoallelic expression.

Based on a wide variety of features (including transcriptome, epigenome and signaling responses), conventional human ESCs are considered to be the human equivalent of mouse EpiSCs [1, 4, 82]. In line, imprinting in human ESCs has been considered to be relatively stable. Recently, various groups have reported the derivation of human ESCs in a naive state akin to mouse ESCs [83–87]. The DNA hypomethylation reported at ICRs in these naive human ESCs suggests that monoallelic expression of imprinted genes is lost, similar to mouse ESCs [88, 89]. However, only few ICRs are well documented, and it has been shown that imprinting can rely on other epigenetic features besides DNA methylation [27, 81, 90]. Therefore, global analysis of imprinted genes by allele-specific RNA-seq in naive human ESCs will be highly informative to further evaluate loss of imprinted gene expression. Also, such profiling will be helpful in the future to further evaluate optimized culture conditions for naive mouse and human ESCs.

## Conclusions

In the current study, we performed allele-specific RNA-seq of early (extra-)embryonic tissues and various types of pluripotent cell lines in vitro using an isogenic mouse F1 genotype, allowing genome-wide comparative analysis of imprinting. This shows that mouse ESCs tend to lose imprinting during outgrowth, while EpiSCs largely retain imprints, even if they are derived from nuclear transfer embryos. Profiling of EpiSC-PGAs shows that known maternally imprinted (paternally expressed) genes are silent, while known paternally imprinted (maternally expressed) genes consistently have a twofold higher expression level in EpiSC-PGAs as compared to their fertilized counterparts. The upregulation of known maternally expressed genes in the EpiSC-PGAs is caused by the absence of the imprinted, silent paternal allele in the EpiSC-PGAs, as these genes show an equal biallelic expression in our allele-specific analysis. Differentiation of ESCs, as well as reversion of ESCs to the pluripotent ground state, does not restore monoallelic expression of imprinted genes, strongly suggesting that the loss of imprinting in ESCs is irreversible. This has important implications for the use of these cells in further applications, as correct imprinting is critical to retain full developmental potential. Together, our analysis provides a comprehensive overview of imprinted gene expression in pluripotency and provides a benchmark to allow identification of pluripotent cell lines that faithfully maintain imprinted gene expression.

## Methods

### Cell culture

Derivation and maintenance of B6D2F1 ESC and EpiSC lines is described in Kobolak et al. [91] and Maruotti et al. [25], respectively. B6D2F1 ESCs were cultured on mouse embryonic fibroblast (MEF) feeder cells in media containing fetal calf serum (FCS). B6D2F1 EpiSCs were maintained feeder-free in chemically defined medium (CDM; [77]) complemented with 20 ng/ml activin A and 12 ng/ml fibroblast growth factor 2 (FGF2), in the absence of fetal bovine serum. In order to get rid of the MEFs for RNA-seq or ChIP-Seq, ESCs were replated on 6 cm feeder-free culture dishes and incubated at 37 °C. After 30 min, floating ESCs were harvested, washed with PBS and collected. An extensive overview of the cell lines used in this study, including sex, passage number, karyotyping, genotype information and names as use previous studies (if the cell lines were derived before) is present in Additional file 1: Table S1. None of the ESC or EpiSC cell lines have tested for their capacity to form teratomas, for their contribution toward chimaeric mice or for their capacity to form completely ES cell-derived fetuses (/germline transmission).

### Embryo sample preparation and RNA-seq

The embryo sample preparation and RNA-seq has been described previously [28]. In short, for preimplantation B6D2F1 mouse embryonic tissues, samples were collected by flushing embryos out of the uterus. For isolation of the E3.5 inner cell mass (ICM) of the expanded blastocyst (Expanded Blastocyst: Inner cell mass; “EBI”), ICMs were isolated from E3.5 B6D2F1 blastocysts by immunosurgery [92]. After removal of the zona pellucida by incubation in acid Tyrode’s solution (Sigma-Aldrich), blastocysts were collected in KSOM (potassium-supplemented simplex optimized medium) containing 20% anti-mouse serum (Sigma-Aldrich) for 30 min at 37 °C. After 3 times washing in KSOM, blastocysts were incubated for 5–10 min in guinea pig complement (Calbiochem-Merck) diluted 1:10 with KSOM. After serial washings, the embryos were cultured for 30–60 min in KSOM. Finally, the ICMs were pipetted through a thin micropipette in order to mechanically remove the lysed trophectoderm cells. For isolation of E3.5 mural trophectoderm (Expanded Blastocyst: mural TrophEctoderm; “EB-TE”), laser-assisted (XY Clone, Hamilton Thorne, UK) ICM isolation was performed on intact, expanded E3.5 blastocysts as previously described [93]. The embryos were secured by two holding pipettes with the ICM being positioned at 9 o’clock. Once adequate tension was established, several (about 10–15) infrared laser pulses (300mW, 1 ms) were fired to section the blastocyst into two uneven portions, of which one consisted of the mural trophectoderm cells.

Tissues from post-implantation stages were obtained as in Brons et al. [1]. Briefly, pregnant female animals were euthanized by cervical dislocation. After surgical removal of the uterine horns, decidua was removed using sharp fine edge forceps. After removing the embryo from the decidua, the Reichert’s membrane was torn out from the embryo and the ectoplacental cone trophoblast tissue was sliced out. The visceral endoderm was removed from the embryo after incubation in cell dissociation buffer (Invitrogen). At E6.25, radially symmetrical epiblast was dissected from the extra-embryonic ectoderm and collected as clean epiblast (Epithelial Radially Symmetrical Epiblast; “ERSE”). At E7.25, the anterior–posterior epiblast was collected with its attached mesodermal wings (Anterior–Posterior Epiblast; “APE”) but without any extraembryonic mesoderm tissues. Samples were harvested into TriZol and snap-frozen. RNA was isolated using the PureLink™ RNA Micro Kit (Invitrogen). 100 pg RNA of the embryo samples was used to prepare cDNA according to the SMARTer (Clontech) method and sequenced paired-end on Illumina Hi-Seq 2000. All RNA-seq of the embryonic tissue samples (FASTQ and BedGraph or wig files) are present in the NCBI GEO SuperSeries GSE53387. An overview of this data is present in Additional file 1: Table S1.

### RNA isolation and cDNA synthesis of ESCs and EpiSCs

RNA was harvested (1) for the ESCs using the RNeasy Plus Mini Kit (QIAGEN) including gDNA eliminator treatment according to instructions of the manufacturer; and (2) for the EpiSCs using TriZol (Invitrogen) according to the manufacturer’s recommendations. Total RNA (100 μg) was subjected to two rounds of poly(A) selection (Oligotex mRNA Mini Kit; QIAGEN), followed by DNaseI treatment (QIAGEN). mRNA (100–200 ng) was fragmented by hydrolysis (5 × fragmentation buffer: 200 mM Tris acetate, pH8.2, 500 mM potassium acetate and 150 mM magnesium acetate) at 94 °C for 90 s and purified (RNAeasy Minelute Kit; QIAGEN). cDNA was synthesized using 5 μg random hexamers by Superscript III Reverse Transcriptase (Invitrogen). Double-stranded cDNA synthesis was performed in second strand buffer (Invitrogen) according to the manufacturer’s recommendations and purified (Minelute Reaction Cleanup Kit; QIAGEN).

### RNA-seq, ChIP-seq and MethylCap-seq of ESCs and EpiSCs

ChIP-Seq was performed according to Marks et al. [50], MethylCap-Seq was performed according to Veillard et al. [94]. For RNA-seq, cDNA was prepared for sequencing either by (1) end repair of 20 ng double-stranded cDNA as measured by Qubit (Invitrogen), ligation of adaptors to DNA fragments followed by size selection (~ 300 bp) and PCR amplification; or by (2) using the Kapa Hyper Prep kit (KAPA Biosystems) according to manufacturer’s protocol, followed by PCR amplification and size selection. Quality control of the adaptor-containing DNA libraries was performed by quantitative PCR and by running the products on an Automated Electrophoresis System [Experion (BioRad) or Bioanalyzer (Agilent)]. Cluster generation and sequencing was performed using the Illumina Genome Analyzer IIx, Hi-Seq 2000 or NextSeq 500 platforms according to standard Illumina protocols. Generation of FASTQ files and demultiplexing was performed using Illumina CASAVA. All sequencing analyses were conducted based on the *M. musculus* NCBI m37 genome assembly (MM9; assembly July 2007). Additional file 1: Table S1 summarizes the sequencing output. All RNA-seq data and ChIP-Seq data (FASTQ and WIG files) are present in the NCBI Gene Expression Omnibus (GEO) SuperSeries GSE101292. An overview of these profiles is present in Additional file 1: Table S1.

### Allele-specific mapping using polymorphic sites between the B6 and DBA2 genomes

Known polymorphic sites between the *Mus musculus domesticus* mouse strains B6 and DBA2 [37] (nucleotide substitutions, not indels) were collected using polymorphic sites determined by (1) the Sanger mouse sequencing project using the March 2011 release using the strains C57BL and DBA [35, 36]; and (2) the NIEHS/Perlegen mouse resequencing project [34] (we used the b04_Chr*_genotype.dat files for the species C57BL/6J (reference genome) and DBA/2J). This resulted in a total of 5,233,965 polymorphic sites between the genomes of B6 and DBA2. FASTQ files were mapped using GSNAP version 2012-07-20 [95]. To avoid bias in the mapping of either the B6- or the DBA2-derived reads, the alternative alleles of polymorphic sites between the B6 and DBA2 genome were included in the reference during mapping (GSNAP SNP-tolerant mapping; flag—v). Only sequence tags aligning to a single position in the genome were included for further analysis. Within the individual samples, we used the mapped tags to determine the coverage per allele for each of the polymorphic sites using GSNAP tally. Per single polymorphic nucleotide, the pile-ups were subsequently assigned to either the B6 or the DBA2 allele using custom Perl-based scripts. Polymorphic sites and analysis for the 129xCast ESCs (Fig. 2b, Additional file 2: Figs. S13 and S14) are described in Marks et al. [42]. Profiles used for the 129xCast ESC analysis were obtained from NCBI GEO SuperSeries GSE60738 [42].

### RNA-seq genotyping

RNA-seq genotyping was performed by determining the percentage of B6 for the sum of B6 or DBA2 allelic counts over all polymorphic sites (B6/(B6 + DBA2) × 100%) in 5 MB or 1 MB chromosomal bins. At 5 MB resolution, we subsequently applied a moving average over four bins for the allelic ratios of bins that contained > 40 counts over polymorphic sites summed for both alleles. Bins containing ≤ 40 SNP counts over polymorphic sites summed for both alleles were labeled “insufficient coverage.” 5 MB bins showing a B6 contribution between 0 and 31% were labeled “DBA2” (paternal; yellow), between 31 and 78.5% “B6/DBA2” (heterozygous; blue) and between 78.5 and 100% “B6” (maternal; red), unless specified otherwise. For the analysis at 1 MB resolution, bins containing > 10 (RNA sequencing) or > 15 (DNA sequencing) counts over polymorphic sites summed for both alleles were included in the analysis. To enable reliable calling of crossover breakpoints, we increased resolution and stringency: 1 MB bins showing a B6 contribution between 0 and 15% were considered DBA2, between 15 and 85% B6/DBA2 heterozygous and between 85 and 100% B6. The genotype at the start of the chromosome proximal to the centromere was assigned B6 or DBA2 if (1) ≥ 80% of 5 bins or (2) ≥ 60% of 10 bins were labeled as either DBA2 or B6; or heterozygous if > 5 consecutive bins were labeled heterozygous. Having characterized the genotype at the proximal part of the centromere, we applied a sliding window approach toward the distal part of the chromosome to estimate crossing-over breakpoints. These breakpoints were identified at positions after which > 5 consecutive bins lacked the label of the assigned genotype (or ≥ 4 consecutive bins of the same genotype at the very proximal end of the chromosome). The genotype present after the breakpoints was determined by applying the same 3 criteria as at the start of the chromosome, or by the ≥ 4 consecutive bins of the same genotype at the proximal end of the chromosome. Breakpoints within 15 MB were discarded if the genotype of the genomic regions neighboring the ≤ 15 MB region was same. The methods as described in this section were also applied for genotyping of the EpiSCs using genomic DNA (obtained from MethylCap-seq experiments present in NCBI GEO SuperSeries GSE47793) [94].

### Gene expression analysis

In light of the difference in sequencing length between the samples (Additional file 1: Table S1), we only included the first 35 bp of the forward read for RPKM quantification, data visualization and PCA/clustering analysis. (In case of paired-end reads, we only included the forward read if the distance between read pairs was < 10 kb to exclude aspecifically mapped reads but to include reads covering introns.) To obtain RNA-seq gene expression values (RPKM), we converted the GSNAP output to Browser Extensible Data (BED) files which were used as input for Genomatix (ElDorado 08–2011) selecting RefSeq genes (NCBI m37 genome assembly; MM9; Additional file 8: Table S7). For viewing, profiles were normalized based on the total number of reads and converted to Wiggle (WIG) files. Differential gene expression was called using DE-Seq 2 applying a Wald test and parametric fitting to estimate dispersion, followed by multiple testing correction using the Benjamini–Hochberg procedure (“adjusted *p* value”), requiring a change > 1.5 fold.

### Calling of allele-specific gene bias

Counts over polymorphic sites within exons of individual RefSeq genes for either B6 or DBA2 were summed to obtain allele-specific gene expression counts for both species (Additional file 8: Table S7). The ratio between the B6 counts or the DBA2 counts versus the total counts (B6 + DBA2) represent the relative contribution of the B6 or DBA2 allele, respectively, to expression of a particular gene. Alternatively, we used the ratio of all allelic counts within genes (log2 B6/DBA2). For analysis of imprinted genes in the various RNA-seq samples, we included genes that (1) contained a coverage of > 9 counts over the polymorphic sites present in the gene; and (2) showed a standard error of the mean (STDEM) of ≤ 0.15 over individual polymorphic sites within a gene (these sites were required to contain a coverage ≥ 3 from either the B6 or DBA2 allele to be included; only applicable if multiple of such polymorphic sites were present). For inclusion in the global allele-specific analysis, we required that individual genes contained coverage over polymorphic sites in each individual ESC, EpiSC or embryonic sample, with a total coverage over polymorphic sites of > 40 for all ESCs or EpiSCs samples and > 200 for all embryonic samples. Allelic bias was defined as > 80% contribution in expression from one allele. Genes showing < 80% allelic bias were considered to be biallelically expressed. For analysis of XCI, we included genes showing a < fourfold allelic bias between B6 and DBA2 in the EpiSC-NT3 line.

### Analysis of epigenetic profiling

ChIP-Seq enriched regions (“peaks”) were determined on tag-normalized BED files using MACS version 1.4.2 at *p* value 10^−6^ for H3K4me3 [96] or, for H3K27me3, using a minimum threshold of 24 overlapping tags, merging enriched regions within 1500 bp. Peak sets of either H3K4me3 or H3K27me3 were merged for all samples and quantified using the tag-normalized BED files by determining the number of reads present within the enriched regions for each of the profiles. Peaks were associated with the closest genes using Pinkthing [97]. For allele-specific quantification of ChIP-Seq peaks, we summed the B6 or BDA2 counts over polymorphic sites within the peaks to calculate the contribution from each allele, similar to the method applied to calculate allelic ratios of gene expression from RNA-seq (see previous sections). Analysis of MethylCap-Seq was performed according to Veillard et al. [94].

## Additional files

**Additional file 1: Table S1.** Overview of the B6D2F1 samples profiled in this study including information on passage number of the cell line, genotyping and allele-specific RNA-Seq and ChIP-Seq.

**Additional file 2: Figure S1.** Validation of the samples included in the current study using regular (non-allele specific) expression analysis of the RNA-Seq. Together with Additional file 2: Fig. S2, this data confirms the developmental stages of the samples. Top panel: Tag-normalized RNA-Seq data over known marker genes for the various pluripotent stages in a genome browser view. Bottom panel: Quantification of expression (RPKM) of the genes present in the genome browser view. The core pluripotency factors are abundantly expressed in most samples, and are higher in the ESCs as compared to the EpiSCs as expected. Known ESC specific factors are highly expressed in ESCs and EBI samples, but largely absent in EpiSCs and ERSE/ APE. Expression of EpiSC-specific markers is largely restricted to EpiSC and ERSE/ APE. Trophectoderm markers are mainly present in the EB-TE samples. **Figure S2.** Validation of developmental stage by principle component analysis (PCA) or clustering using global quantile normalized RPKM expression values (log2). We included genes showing a total RPKM >2 in ESCs and EpiSCs (16,059 genes out of 21,345 RefSeq genes). **(a)** PCA analysis showing a clear separation of samples along the first two principle components. The first principle component (PC; x-axis), explaining 26% of the total variation separates the *in vivo* versus the *in vitro* samples. This PC also includes the variation introduced during the library preparation of the RNA-Seq, as the *in vivo* samples are prepared by the low-input polyA-based SMARTer RNA-Seq method containing an amplification step while the ESC and EpiSC samples are prepared by regular polyA-selected RNA-Seq. The second principle component (y-axis), explaining 19% of the variation, mainly separates early from late embryonic stages for both the *in vivo* and *in vitro* samples. **(b)** Heatmap of correlation (Pearson’s r) including clustering using Euclidean distance showing a clear separation of the various cell types. **Figure S3.** Genotype of ESC lines as determined by RNA-Seq genotyping at 5MB resolution. The horizontal axis represents chromosomes, the vertical axis chromosomal bins (per 5 MB). The numbers within each bin (also categorized by the three colors) represent the percentage B6 as compared to the total coverage of B6 and DBA2 over the SNPs in each bin. The ESC lines are female unless indicated otherwise. X0: female ESCs with only a single X chromosome. **Figure S4.** Genotype of the embryonic tissues included in the current study as determined by RNA-Seq genotyping at 5MB resolution. See legend Additional file 2: Fig. S3 for further details. **Figure S5.** Genotype of EpiSC lines as determined by RNA-Seq genotyping at 5MB resolution. See legend Additional file 2: Fig. S3 for further details. The EpiSC lines are female unless indicated otherwise. The allelic bias observed for the X chromosome in EpiSC1, EpiSC-NT1 and EpiSC-NT2 is further discussed in Fig. 4 and the corresponding main text. **Figure S6.** Genotype of the EpiSC lines EpiSC-PGA1, EpiSC-PGA2 and EpiSC-NT1 based on genomic sequencing at 5MB resolution. See legend Additional file 2: Fig. S3 for further details. **Figure S7.** Validation of the RNA-Seq genotyping of the EpiSC-PGAs. Distribution of relative expression from the B6 versus the DBA2 allele of the genes present within genomic regions genotyped as either homozygous B6 (red), heterozygous B6/DBA2 (blue) or homozygous DBA2 (yellow) in the EpiSC-PGAs. A log2 ratio of 0 represents equal biallelic gene expression from the B6 and DBA2 alleles, while positive and negative ratios represent higher expression from the B6 or DBA2 allele, respectively. Genes present in the part of the genome genotyped as heterozygous are largely expressed from both alleles, while alleles of genes present in the homozygous part of the genome cannot be discriminated (and therefore these genes show a (near) complete bias according to their genotype). **Figure S8.** Genotype of EpiSC2 line as determined by regular genotyping or RNA-Seq based genotyping at 5MB resolution. The horizontal axis represents chromosomes, the vertical axis chromosomal bins (per 5 MB). The numbers within each bin (also categorized by five colors) represent the percentage B6 as compared to the total coverage of B6 and DBA2 over the SNPs. The allelic bias as obtained for chromosome 18 (~30% DBA2 and ~70% B6) suggests the presence of a trisomy of chromosome 18 (two copies of DBA2, one copy of B6). **Figure S9.** Distribution of relative gene expression from the B6 versus the DBA2 allele in the B6D2F1 samples over autosomes, showing that the majority of genes have an equal expression from the B6 and DBA2 allele. A log2 ratio of 0 represents equal biallelic gene expression from the B6 and DBA2 alleles, while positive and negative ratios represent higher expression from the B6 or DBA2 allele, respectively. On top the number of genes included in each of the boxplots. We obtained quantitative allelic information for up to 3,110 genes for the embryonic tissues, and up to 3,998 or 4,995 genes for the ESCs and EpiSCs, respectively (out of a total of 21,345 unique RefSeq genes). For the ESC-PGA and EpiSC-PGA lines, for which our analysis is restricted to the heterozygous B6/ DBA2 parts of the genome as identified in Additional file 3: Table S2 and Additional file 4: Table S3, we obtained allele-specific quantification for between 2,514-2,994 genes (dependent on the line). The larger spread of allelic ratios as present in the embryonic tissues is likely due to the amplification procedure necessary during construction of the RNA-Seq library for the very small amounts of RNA obtained from the embryonic tissues. **Figure S10:** Example of the tag-normalized RNA-Seq data over a selection of imprinted genes as included in this study. **Figure S11.** Validation of the H3K4me3 and H3K27me3 ChIP-Seq performed on 3 B6D2F1 ESCs lines. Example of tag-normalized ChIP-Seq data over known pluripotency markers as well as imprinted genes. For the highly expressed pluripotency markers, we detect clear H3K4me3 peaks on the promoters, but no H3K27me3, as expected. Dependent on the gene, the imprinted genes contain promoter-associated H3K4me3 (active), H3K27me3 (silent) or both (bivalent; associated with low level of expression) [48]. **Figure S12.** (Allelic) epigenetic landscape of fertilized ESC, ESC-NT and ESC-PGA lines, showing that the majority of the H3K4me3 and/or H3K27me3 enriched loci associated with imprinted loci are equally present at both alleles. **(a)** Allelic bias of H3K4me3 or H3K27me3 enriched loci (ChIP-Seq “peaks”) associated with known imprinted genes plotted by the percentage of B6 as compared to the total coverage of B6 and DBA2 per peak. Within both individual panels, the left axis indicates that the peak is largely present on the DBA2 (paternal) allele, the right axis indicates that the peak is largely present on the B6 (maternal) allele, the middle axis indicated that the peak is equally present on both alleles. The left panel visualizes H3K4me3, the right panel represents H3K27me3. The graph only includes data points if (i) a gene was associated with a peak for H3K4me3 or H3K27me3 and (ii) the peak contained SNPs to discriminate between the DBA2 and B6 allele. Since only a minority of the imprinted genes shown are associated with H3K27me3 (see Additional file 2: Fig. S11), the H3K27me3 panel contains relatively few data points. “P” = paternally expressed; “M” = maternally expressed. **(b)** Quantification of ChIP-Seq peaks shown in panel (a). This panel additionally includes peaks that do not contain SNPs to determine allelic bias and could therefore not be included in panel (a). **Figure S13.** Allelic bias in expression of known imprinted genes as shown in Fig. 2a plotted by the percentage of B6 as compared to the total coverage of B6 and DBA2 (left) or the percentage of 129 as compared to the total coverage of 129 and Cast (right). Within both individual panels, the left axis represents expression from the paternal allele, the right axis represents expression from the maternal allele, the middle axis represents equal biallelic expression. The graph only includes data points of genes for which we obtained sufficient coverage to calculate allelic bias, explaining the variable number of data points between genes or the complete lack of data points for some genes in either of the cell lines. The left panel (data points in red) visualizes B6D2F1 ESC lines and is the same as shown in Fig. 2a. The individual replicas of all samples are included in the graph, but not individually labeled. The right panel (data points in gray and black) represents 129xCast ESC lines either derived and maintained in the presence of serum and LIF (black; 129CastF1) or adapted to 2i + LIF (gray; 129CastF1_2i) [42]. The 129CastF1 ESCs are previously referred to as ES_Tsix-stop [42]. “P” = paternally expressed; “M” = maternally expressed. **Figure S14.** Similar to Additional file 2: Fig. S13, showing allelic expression of imprinted genes during embryoid body (EB) differentiation of ESCs maintained in 2i+LIF (left; the same as Fig. 2b) or serum+LIF (right panel). **Figure S15.** Quantification of expression levels (RPKM) of the genes shown in Fig. 2a.This figure matches Fig. 2c, but additionally includes quantification of gene expression of B6D2F1 embryonic tissues. **Figure S16.** Quantification of expression levels (RPKM) of Rian and Meg3, as well as Igf2 and Cdkn1c, representing imprinted genes misregulated in (a selection of) B6D2F1 EpiSC-NT lines. **Figure S17.** DNA methylation analysis of Imprinted Control Regions (ICRs). Genome-wide DNA methylation profiles were generated using MethylCap-Seq. The four profiles were normalized to 7,139,891 sequence tags to allow quantitative comparisons. ICR coordinates were obtained from Mikkelsen et al. [98] and Ferguson-Smith [99]. **(a)** DNA methylation over known ICRs in a genome browser view. For clarity, some of the ICRs are boxed. For the imprinted maternally expressed genes (ICR on paternal allele methylated), both PGA lines show a loss of DNA methylation as compared to the fertilized EpiSCs as expected for PGA lines. For the imprinted paternally expressed genes (ICR on maternal allele methylated), both PGA lines show ~2 fold increase in DNA methylation as compared to the fertilized EpiSCs as expected for PGA lines. Notably, for the ICRs for which there is one or more polymorphic site(s) to discriminate alleles, both EpiSC1 and EpiSC2 show the anticipated, monoallelic presence of DNA methylation on the expected allele (for Rasgrf1 (paternal) and Snrpn, Mcts2/ H13, Sgce/ Peg10, Plagl1 and Impact (all maternal) (data not shown)). **(b)** Quantification (on tag counts) of peaks as shown in panel (a), including the fold change of the peak in the EpiSC-PGAs as compared to the fertilized EpiSCs (yellow header; green indicates decrease of peak, red indicates increase of peak). **Figure S18.** B6/DBA2 ratio per gene over the linear X chromosome (the X-axis representing genomic coordinates in MB) ) in ESCs **(a)** or EpiSCs **(b)**, similar to Fig. 4d. Each dot represents a gene. In blue the B6/DBA2 ratio obtained by DNA sequencing at 5MB resolution, confirming the presence of a B6 and DBA chromosome X in the female EpiSC1 and EpiSC-NT1 lines and the presence of a single B6 X chromosome in the male EpiSC2 and EpiSC3 lines. XIC = X inactivation center.

**Additional file 3: Table S2.** Genotype and breakpoints of the B6D2F1 ESC-PGA lines used in this study as determined by RNA-Seq at 1 MB resolution. Red represents B6 on both alleles, blue represent heterozygous B6D2F1, yellow represents DBA2 on both alleles. A shift in genotype, indicated with a “,”, represents a recombination breakpoint that occurred in the B6D2F1 oocyte.

**Additional file 4: Table S3.** Genotype and breakpoints of the B6D2F1 EpiSC-PGA lines as determined by genomic sequencing or RNA-Seq at 1 MB resolution. Red represents B6 on both alleles, blue represent heterozygous B6D2F1, yellow represents DBA2 on both alleles. A shift in genotype, indicated with a “,”, represents a recombination breakpoint that occurred in the B6D2F1 oocyte. The RNA-Seq genotyping algorithm identifies the correct position of the genomic breakpoints in 47% (9 of 19) and 64% (14 of 22) of the cases in EpiSC-PGA1 and EpiSC-PGA2, respectively, with an average accuracy of 1.3MB (EpiSC-PGA1) and 4.6MB (EpiSC-PGA2). The lower accuracy in EpiSC-PGA2 is mainly because three of the breakpoints are present in gene-poor loci where the RNA-Seq-based genotyping method lacks resolution. The distribution of relative expression from the B6 versus the DBA2 allele of the genes present within genomic regions genotyped as either homozygous B6, heterozygous B6/DBA2 or homozygous DBA2 of both EpiSC-PGA lines is present in Additional file 2: Fig. S7.

**Additional file 5: Table S4.** Compiled list of imprinted genes.

**Additional file 6: Table S5.** Allelic bias in expression of known imprinted genes shown as percentage of B6 as compared to the total coverage of B6 and DBA2 per gene. The table below includes gene expression values, read counts, allelic counts and standard error of the mean of the imprinted genes. For the upper part, the table only includes data points of genes for which we obtained sufficient coverage to calculate allelic bias, explaining the empty cells in the table.

**Additional file 7: Table S6.** Differential gene expression between fertilized EpiSCs versus EpiSC-NTs or EpiSC-PGAs (adjusted p-value <0.05).

**Additional file 8: Table S7.** Genome-wide gene expression values, allelic counts and standard error of the mean for the RNA-Seq profiles generated in this study.
