# Supplementary material for: Allele-specific RNA-seq expression profiling of imprinted genes in mouse isogenic pluripotent states
Source: Epigenetics Chromatin. 2019 Feb 15;12:14. doi: 10.1186/s13072-019-0259-8 (PMC6376749; doi:10.1186/s13072-019-0259-8)
Supplement: Supplementary file 2 — Additional file 2: Figure S1. Validation of the samples included in the current study using regular (non-allele specific) expression analysis of the RNA-Seq. Together with Additional file 2: Fig. S2, this data confirms the developmental stages of the samples. Top panel: Tag-normalized RNA-Seq data over known marker genes for the various pluripotent stages in a genome browser view. Bottom panel: Quantification of expression (RPKM) of the genes present in the genome browser view. The core pluripotency factors are abundantly expressed in most samples, and are higher in the ESCs as compared to the EpiSCs as expected. Known ESC specific factors are highly expressed in ESCs and EBI samples, but largely absent in EpiSCs and ERSE/ APE. Expression of EpiSC-specific markers is largely restricted to EpiSC and ERSE/ APE. Trophectoderm markers are mainly present in the EB-TE samples. Figure S2. Validation of developmental stage by principle component analysis (PCA) or clustering using global quantile normalized RPKM expression values (log2). We included genes showing a total RPKM >2 in ESCs and EpiSCs (16,059 genes out of 21,345 RefSeq genes). (a) PCA analysis showing a clear separation of samples along the first two principle components. The first principle component (PC; x-axis), explaining 26% of the total variation separates the in vivo versus the in vitro samples. This PC also includes the variation introduced during the library preparation of the RNA-Seq, as the in vivo samples are prepared by the low-input polyA-based SMARTer RNA-Seq method containing an amplification step while the ESC and EpiSC samples are prepared by regular polyA-selected RNA-Seq. The second principle component (y-axis), explaining 19% of the variation, mainly separates early from late embryonic stages for both the in vivo and in vitro samples. (b) Heatmap of correlation (Pearson’s r) including clustering using Euclidean distance showing a clear separation of the various cell types. Figure S3. Genotyp [file 13072_2019_259_MOESM2_ESM.pdf]

Additional file 2: Supplementary figures belonging to: Allele-specific RNA-Seq expression profiling of imprinted genes in mouse isogenic pluripotent states, by Dirks et al. (2019), Epigenetics & Chromatin

Figure S1

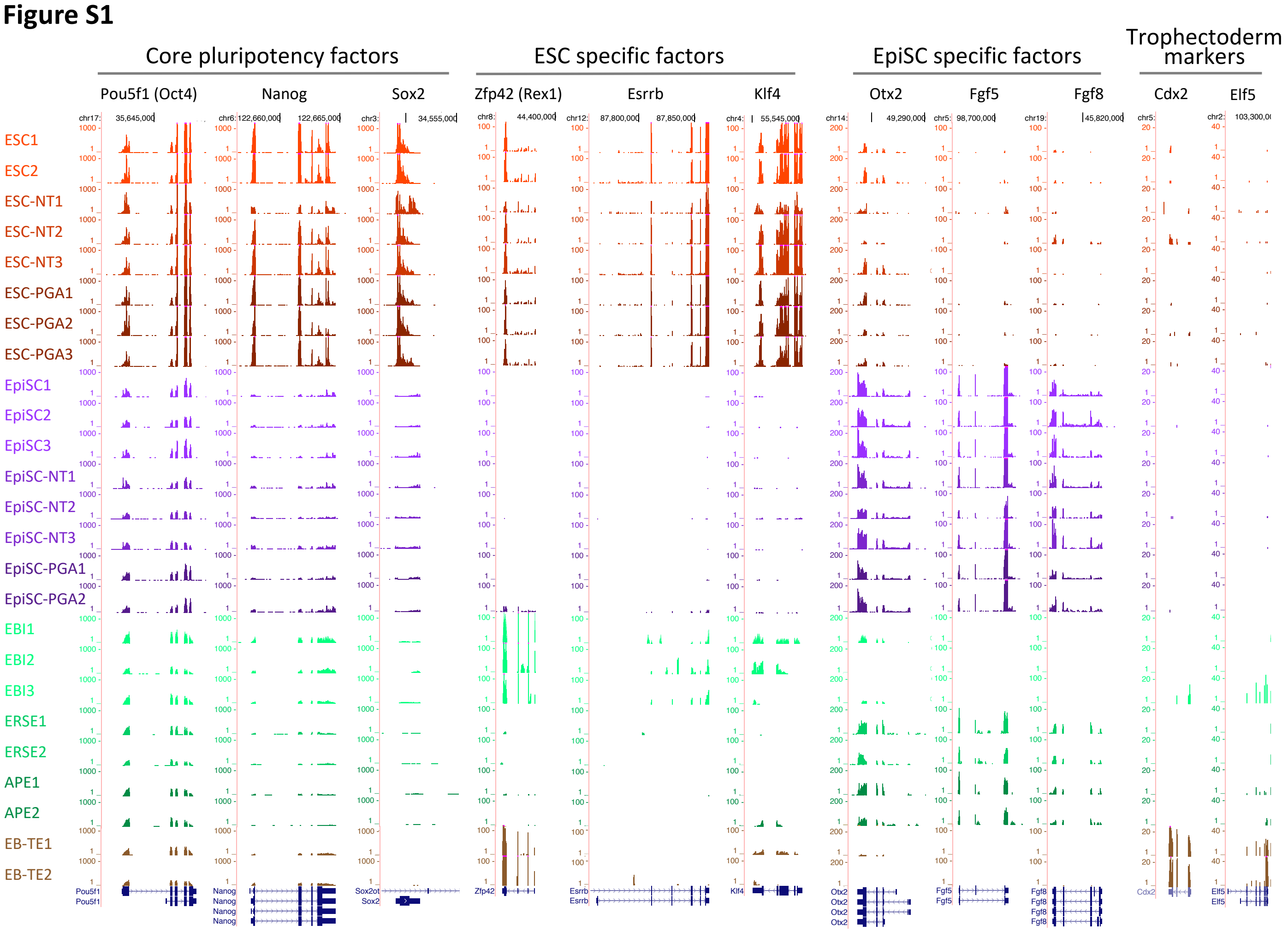

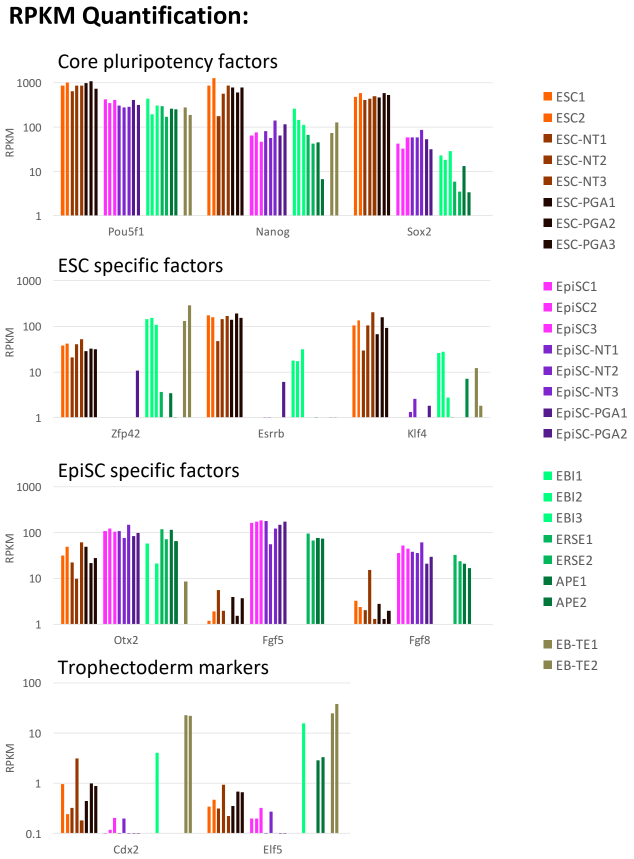

Figure S1. Validation of the samples included in the current study using regular (non-allele specific) expression analysis of the RNA-Seq. Together with Additional File 2: Fig. S2, this data confirms the developmental stages of the samples. **Top panel:** Tag-normalized RNA-Seq data over known marker genes for the various pluripotent stages in a genome browser view. **Bottom panel:** Quantification of expression (RPKM) of the genes present in the genome browser view. The core pluripotency factors are abundantly expressed in most samples, and are higher in the ESCs as compared to the EpiSCs as expected. Known ESC specific factors are highly expressed in ESCs and EBI samples, but largely absent in EpiSCs and ERSE/ APE. Expression of EpiSC-specific markers is largely restricted to EpiSC and ERSE/ APE. Trophectoderm markers are mainly present in the EB-TE samples.

Figure S2

Fig. S2a

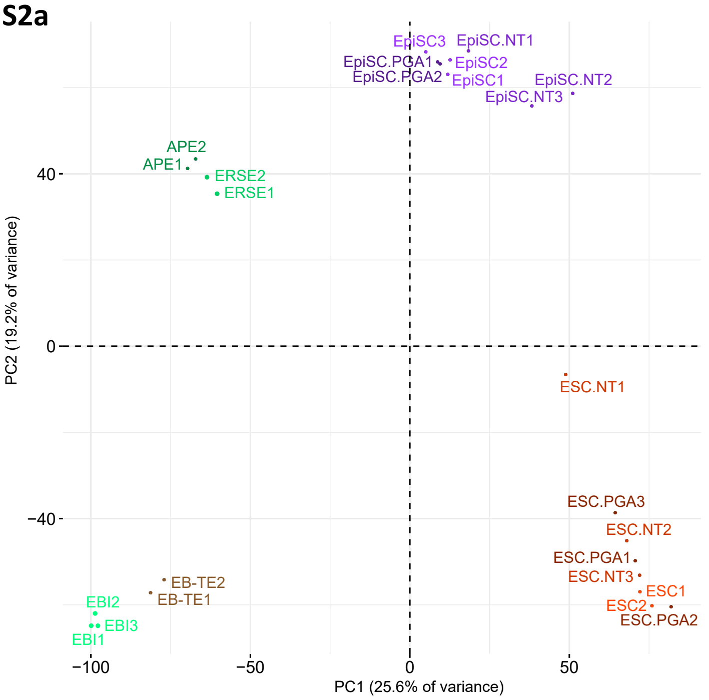

Fig. S2b

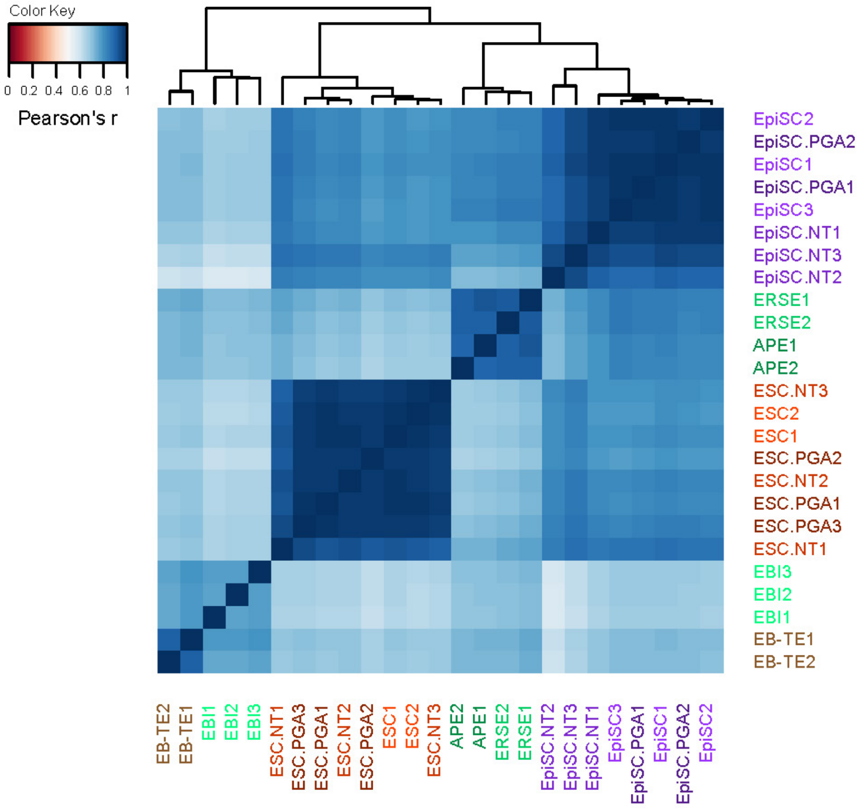

Figure S2. Validation of developmental stage by principle component analysis (PCA) or clustering using global quantile normalized RPKM expression values (log2). We included genes showing a total RPKM >2 in ESCs and EpiSCs (16,059 genes out of 21,345 RefSeq genes).

(a) PCA analysis showing a clear separation of samples along the first two principle components. The first principle component (PC; x-axis), explaining 26% of the total variation separates the *in vivo* versus the *in vitro* samples. This PC also includes the variation introduced during the library preparation of the RNA-Seq, as the *in vivo* samples are prepared by the low-input polyA-based SMARTer RNA-Seq method containing an amplification step while the ESC and EpiSC samples are prepared by regular polyA-selected RNA-Seq. The second principle component (y-axis), explaining 19% of the variation, mainly separates early from late embryonic stages for both the *in vivo* and *in vitro* samples. **(b)** Heatmap of correlation (Pearson's r) including clustering using Euclidean distance showing a clear separation of the various cell types.

Figure S3

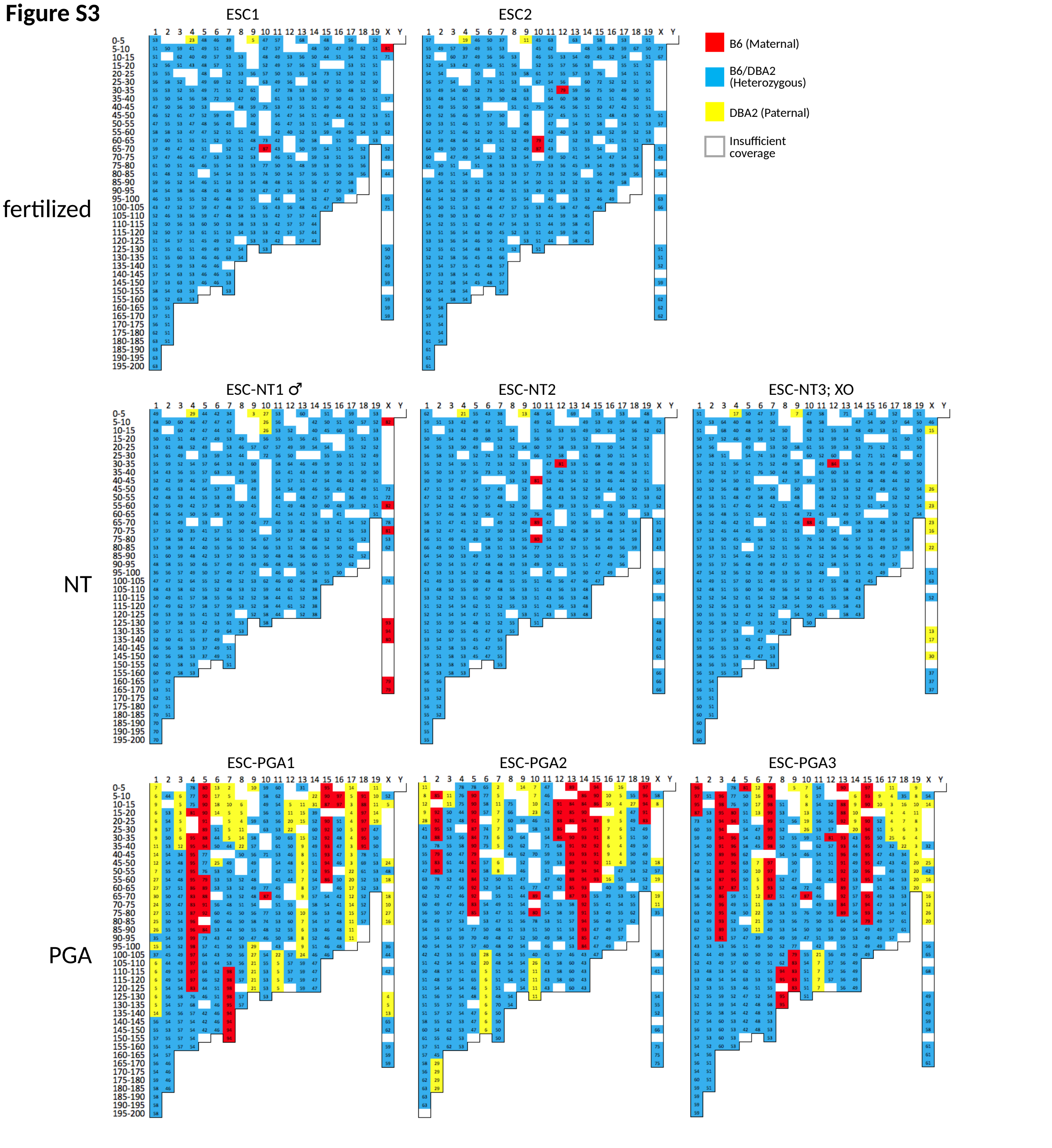

Figure S3. Genotype of ESC lines as determined by RNA-Seq genotyping at 5MB resolution. The horizontal axis represents chromosomes, the vertical axis chromosomal bins (per 5 MB). The numbers within each bin (also categorized by the three colors) represent the percentage B6 as compared to the total coverage of B6 and DBA2 over the SNPs in each bin. The ESC lines are female unless indicated otherwise. X0: female ESCs with only a single X chromosome.

Figure S4

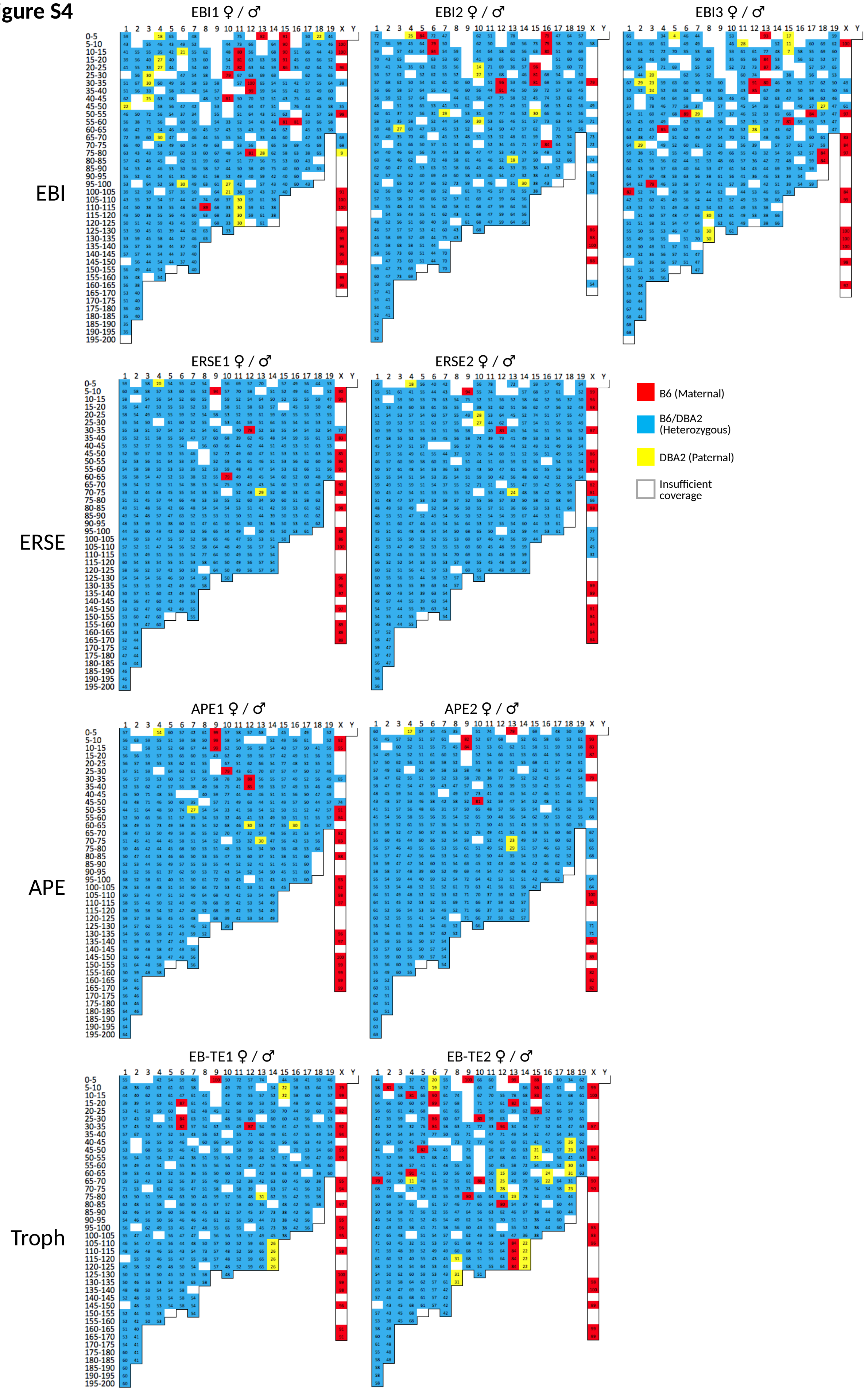

Figure S4. Genotype of the embryonic tissues included in the current study as determined by RNA-Seq genotyping at 5MB resolution. See legend Additional file 2: Fig. S3 for further details.

Figure S5

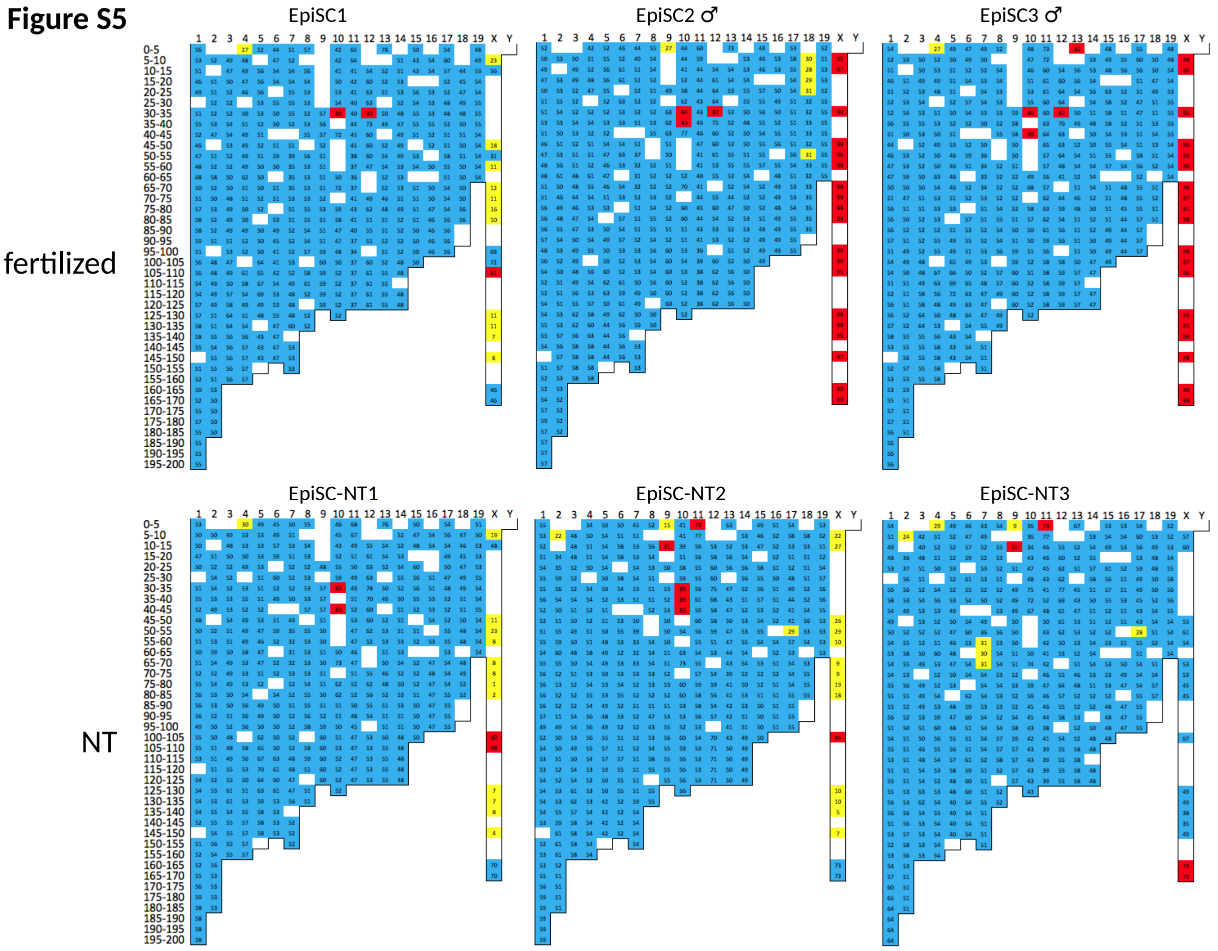

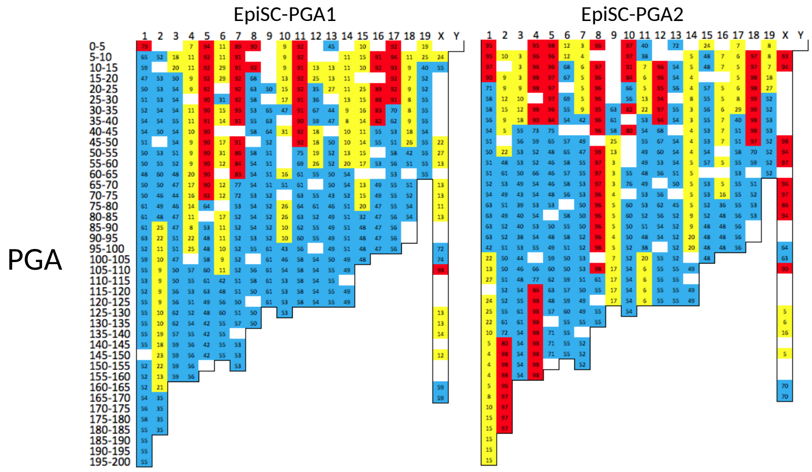

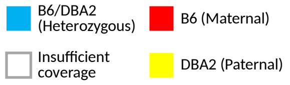

Figure S5. Genotype of EpiSC lines as determined by RNA-Seq genotyping at 5MB resolution. See legend Additional file 2: Fig. S3 for further details. The EpiSC lines are female unless indicated otherwise. The allelic bias observed for the X chromosome in EpiSC1, EpiSC-NT1 and EpiSC-NT2 is further discussed in Fig. 4 and the corresponding main text.

Figure S6 Genomic sequencing

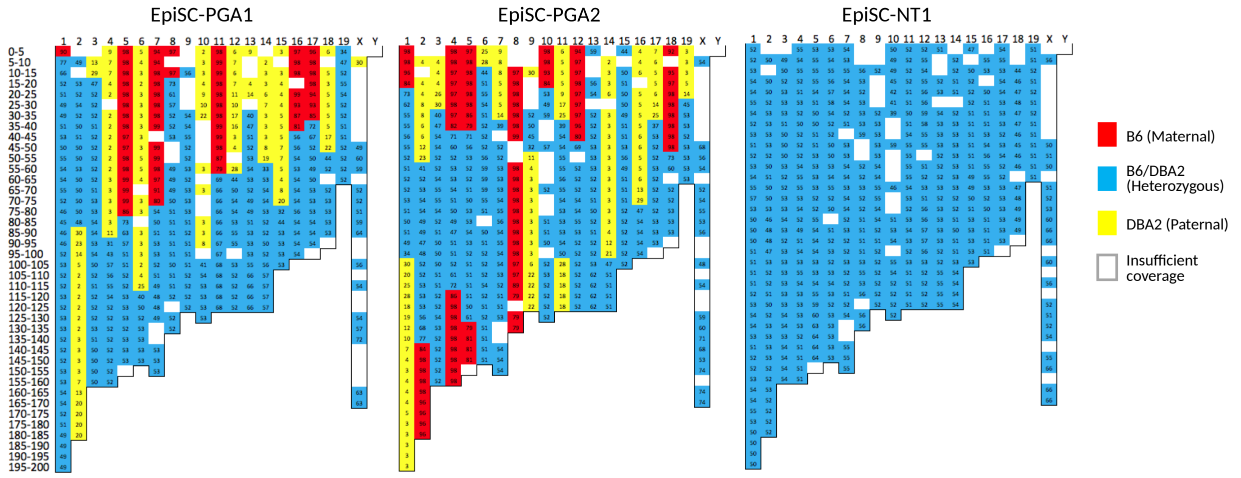

Figure S6. Genotype of the EpiSC lines EpiSC-PGA1, EpiSC-PGA2 and EpiSC-NT1 based on genomic sequencing at 5MB resolution. See legend Additional file 2: Fig. S3 for further details.

Figure S7

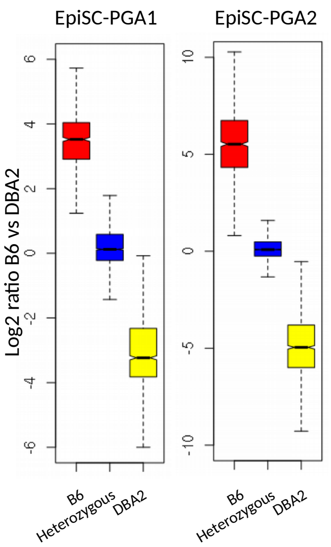

Figure S7. Validation of the RNA-Seq genotyping of the EpiSC-PGAs. Distribution of relative expression from the B6 versus the DBA2 allele of the genes present within genomic regions genotyped as either homozygous B6 (red), heterozygous B6/DBA2 (blue) or homozygous DBA2 (yellow) in the EpiSC-PGAs. A log2 ratio of 0 represents equal biallelic gene expression from the B6 and DBA2 alleles, while positive and negative ratios represent higher expression from the B6 or DBA2 allele, respectively. Genes present in the part of the genome genotyped as heterozygous are largely expressed from both alleles, while alleles of genes present in the homozygous part of the genome cannot be discriminated (and therefore these genes show a (near) complete bias according to their genotype).

Figure S8

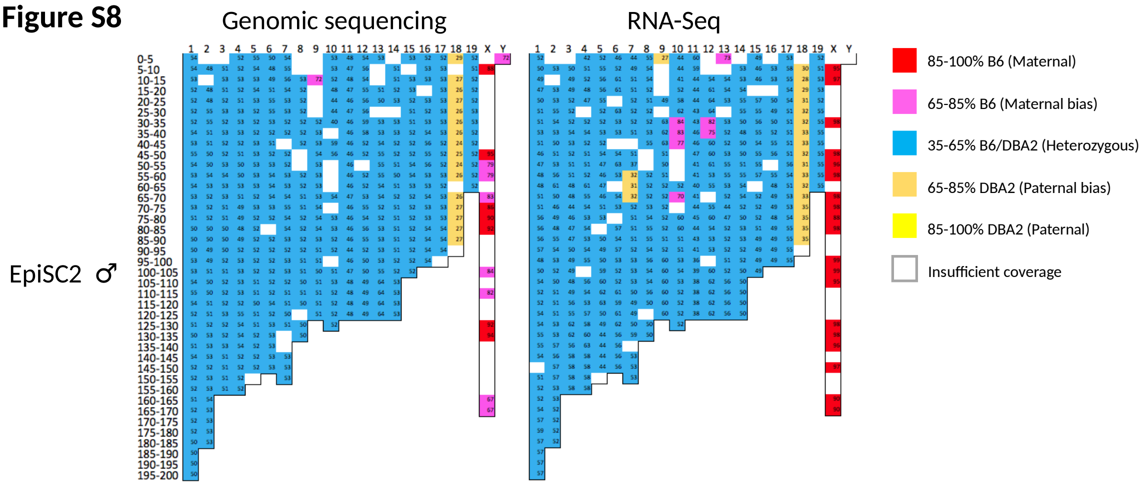

Figure S8. Genotype of EpiSC2 line as determined by regular genotyping or RNA-Seq based genotyping at 5MB resolution. The horizontal axis represents chromosomes, the vertical axis chromosomal bins (per 5 MB). The numbers within each bin (also categorized by five colors) represent the percentage B6 as compared to the total coverage of B6 and DBA2 over the SNPs. The allelic bias as obtained for chromosome 18 (~30% DBA2 and ~70% B6) suggests the presence of a trisomy of chromosome 18 (two copies of DBA2, one copy of B6).

Figure S9

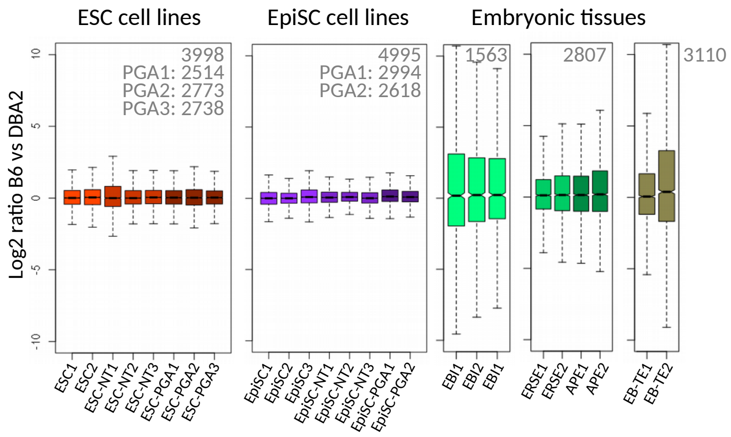

Figure S9. Distribution of relative expression of genes from the B6 versus the DBA2 allele in the B6D2F1 samples over autosomes, showing that the majority of genes have an equal expression from the B6 and DBA2 allele. A log2 ratio of 0 represents equal biallelic gene expression from the B6 and DBA2 alleles, while positive and negative ratios represent higher expression from the B6 or DBA2 allele, respectively. On top the number of genes included in each of the boxplots. We obtained quantitative allelic information for up to 3,110 genes for the embryonic tissues, and up to 3,998 or 4,995 genes for the ESCs and EpiSCs, respectively (out of a total of 21,345 unique RefSeq genes). For the ESC-PGA and EpiSC-PGA lines, for which our analysis is restricted to the heterozygous B6/ DBA2 parts of the genome as identified in Additional file 3: Table S2 and Additional file 4: Table S3, we obtained allele-specific quantification for between 2,514-2,994 genes (dependent on the line). The larger spread of allelic ratios as present in the embryonic tissues is likely due to the amplification procedure necessary during construction of the RNA-Seq library for the very small amounts of RNA obtained from the embryonic tissues.

Figure S10

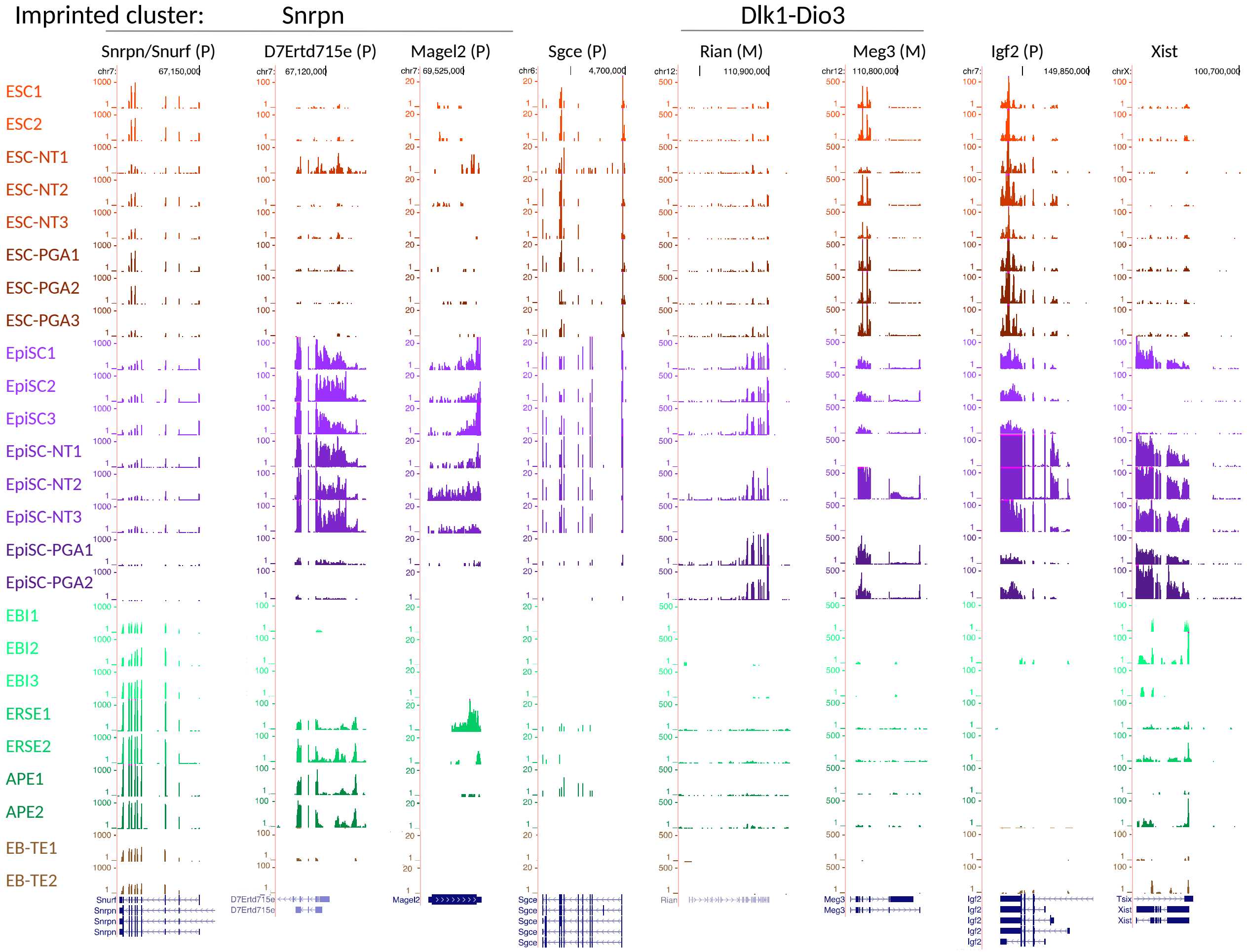

Figure S10: Example of the tag-normalized RNA-Seq data over a selection of imprinted genes as included in this study.

Figure S11 ESC cell lines

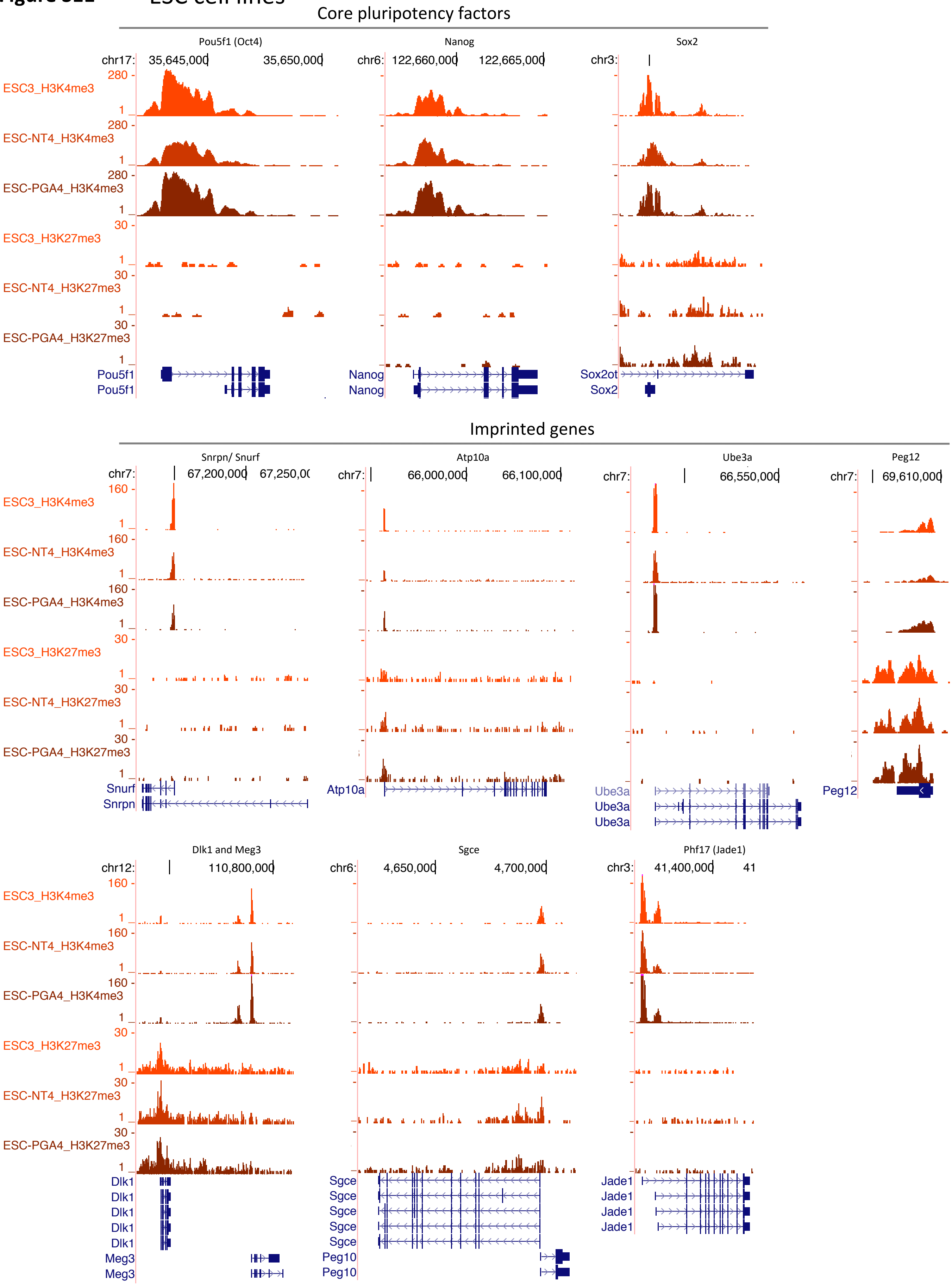

Figure S11. Validation of the H3K4me3 and H3K27me3 ChIP-Seq performed on three B6D2F1 ESCs lines. Example of tag-normalized ChIP-Seq data over known pluripotency markers as well as imprinted genes. For the highly-expressed pluripotency markers, we detect clear H3K4me3 peaks on the promoters, but no H3K27me3, as expected. Dependent on the gene, the imprinted genes contain promoter-associated H3K4me3 (active), H3K27me3 (silent) or both (bivalent; associated with low level of expression; Bernstein et al. [48]).

Figure S12

Fig. S12a

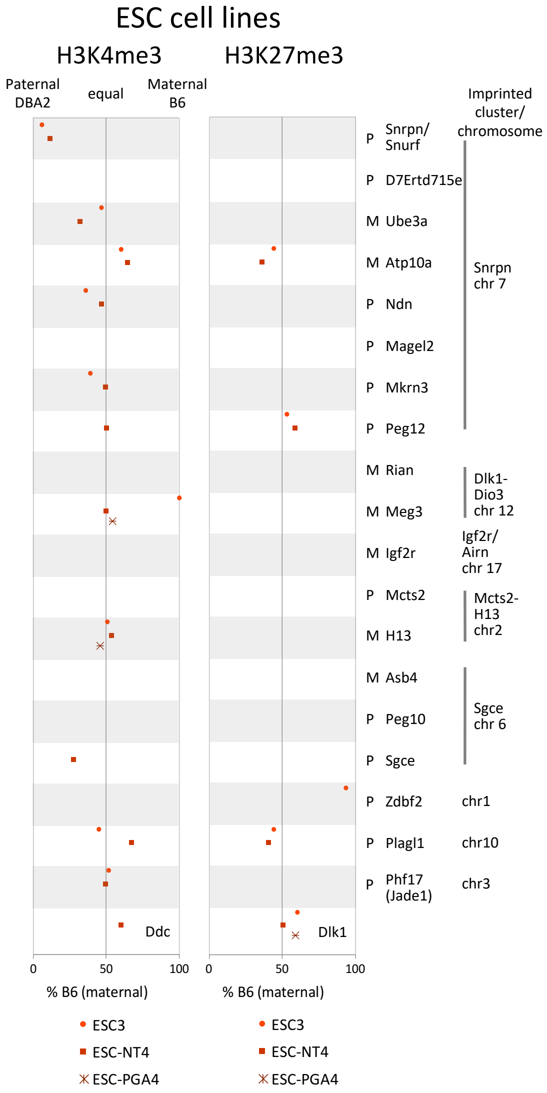

Fig. S12b

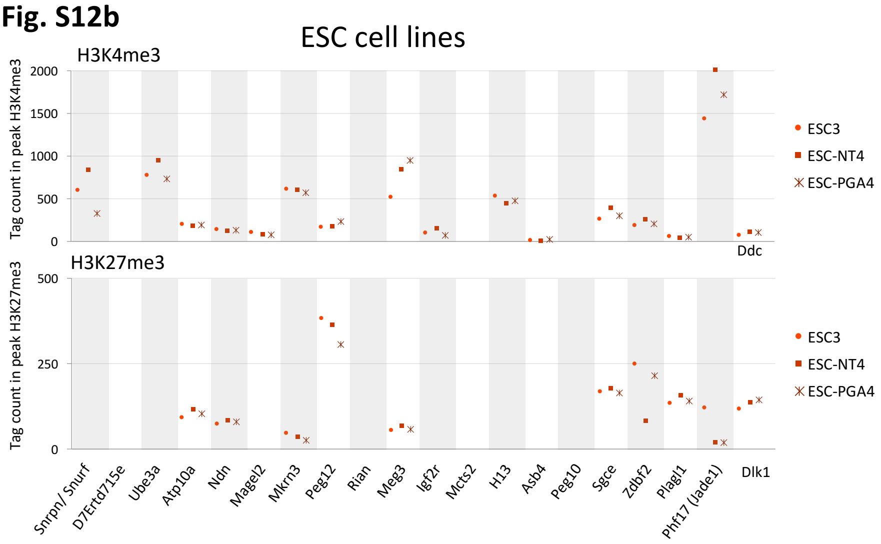

Figure S12. (Allelic) epigenetic landscape of fertilized ESC, ESC-NT and ESC-PGA lines, showing that the majority of the H3K4me3 and/or H3K27me3 enriched loci associated with imprinted loci are equally present at both alleles.

(a) Allelic bias of H3K4me3 or H3K27me3 enriched loci (ChIP-Seq “peaks”) associated with known imprinted genes plotted by the percentage of B6 as compared to the total coverage of B6 and DBA2 per peak. Within both individual panels, the left axis indicates that the peak is largely present on the DBA2 (paternal) allele, the right axis indicates that the peak is largely present on the B6 (maternal) allele, the middle axis indicated that the peak is equally present on both alleles. The left panel visualizes H3K4me3, the right panel represents H3K27me3. The graph only includes data points if (i) a gene was associated with a peak for H3K4me3 or H3K27me3 and (ii) the peak contained SNPs to discriminate between the DBA2 and B6 allele. Since only a minority of the imprinted genes shown are associated with H3K27me3 (see Additional file 2: Fig. S11), the H3K27me3 panel contains relatively few data points. “P” = paternally expressed; “M” = maternally expressed. **(b)** Quantification of ChIP-Seq peaks shown in panel (a). This panel additionally includes peaks that do not contain SNPs to determine allelic bias and could therefore not be included in panel (a).

Figure S13

ESC cell lines

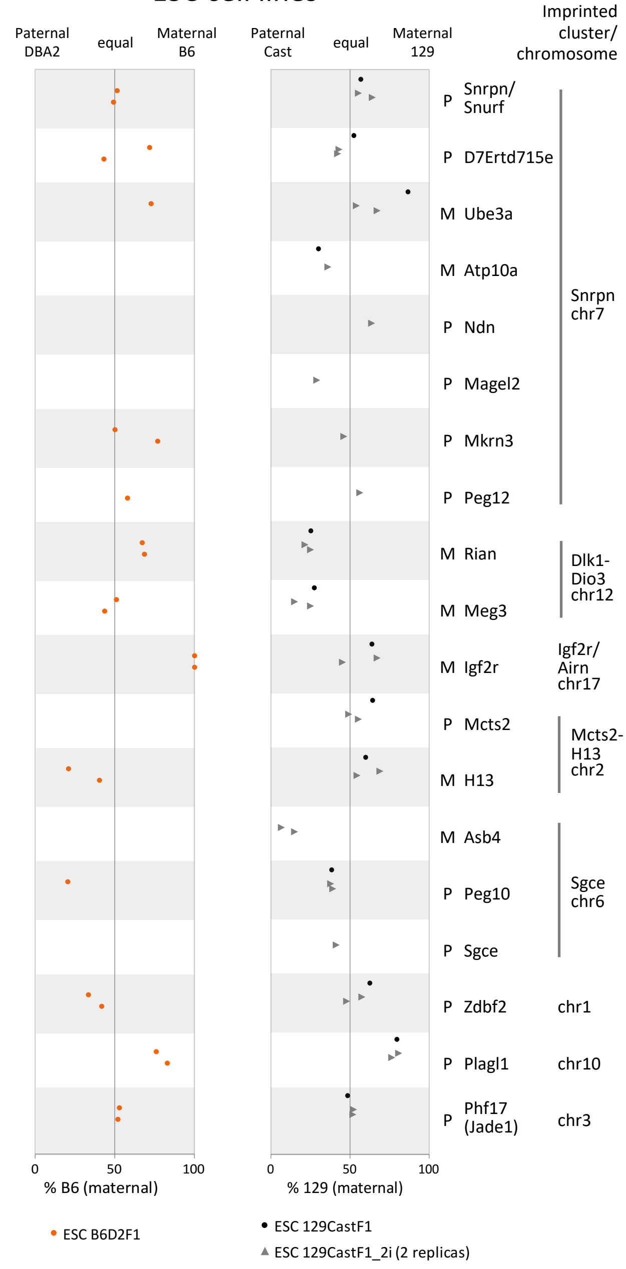

Figure S14

ESC cell line differentiation

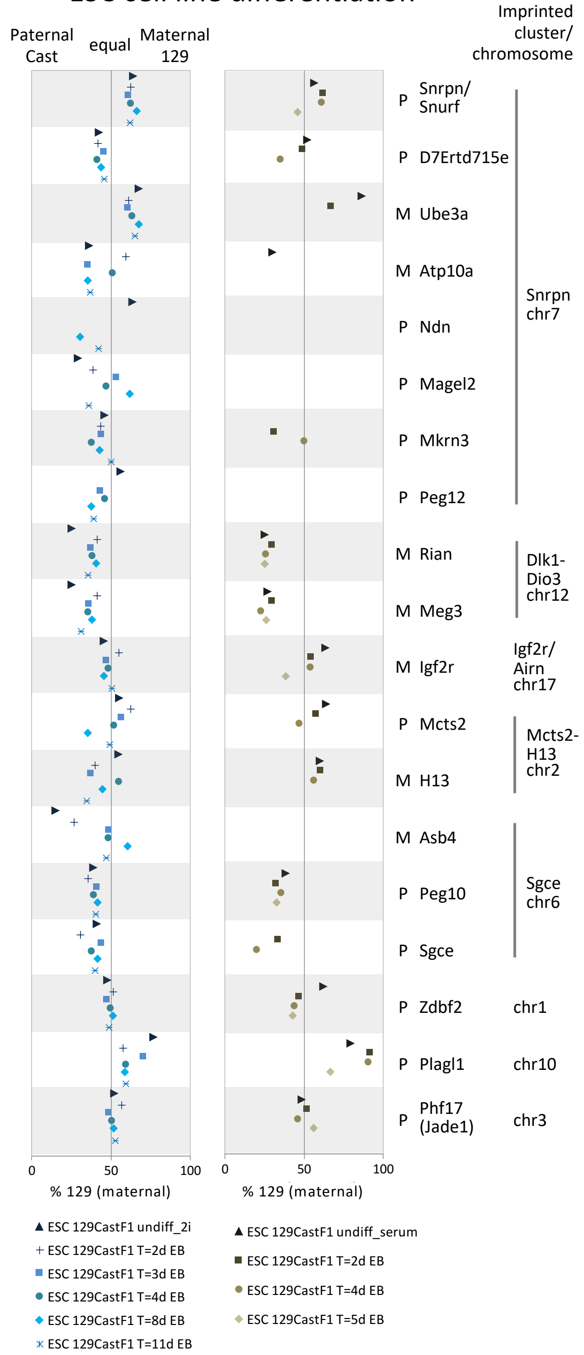

Figure S13. Allelic bias in expression of known imprinted genes as shown in Fig. 2a plotted by the percentage of B6 as compared to the total coverage of B6 and DBA2 (left) or the percentage of 129 as compared to the total coverage of 129 and Cast (right). Within both individual panels, the left axis represents expression from the paternal allele, the right axis represents expression from the maternal allele, the middle axis represents equal biallelic expression. The graph only includes data points of genes for which we obtained sufficient coverage to calculate allelic bias, explaining the variable number of data points between genes or the complete lack of data points for some genes in either of the cell lines. The left panel (data points in red) visualizes B6D2F1 ESC lines and is the same as shown in Fig. 2a. The individual replicas of all samples are included in the graph, but not individually labeled. The right panel (data points in gray and black) represents 129xCast ESC lines either derived and maintained in the presence of serum and LIF (black; 129CastF1) or adapted to 2i + LIF (gray; 129CastF1_2i) (Marks et al., [42]). The 129CastF1 ESCs are previously referred to as ES_Tsix-stop (Marks et al., [42]). “P” = paternally expressed; “M” = maternally expressed.

Figure S14. Similar to Additional file 2: Fig. S13, showing allelic expression of imprinted genes during embryoid body (EB) differentiation of ESCs maintained in either 2i+LIF (left panel; the same as main Fig. 2b) or serum+LIF (right panel).

Figure S15

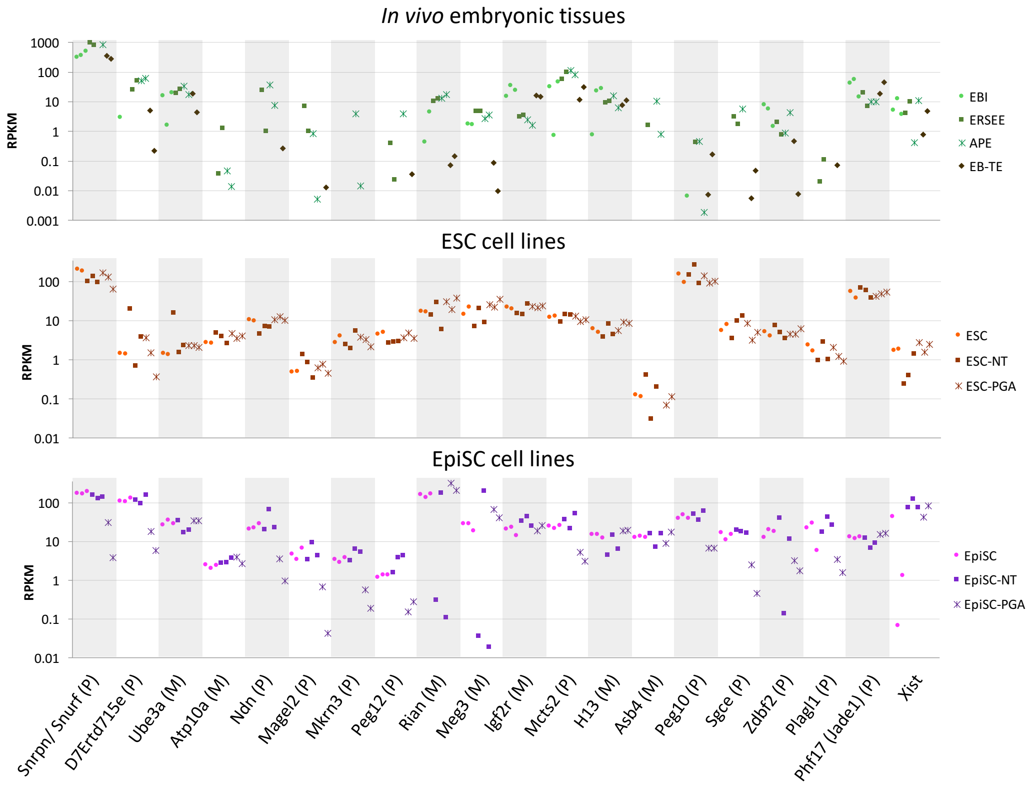

Figure S15. Quantification of expression levels (RPKM) of the genes shown in Fig. 2a. This figure matches Fig. 2c, but additionally includes quantification of expression levels of the B6D2F1 embryonic tissues.

Figure S16

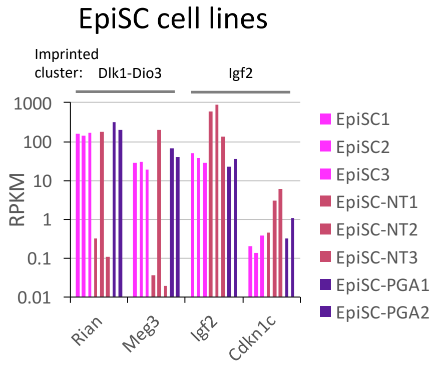

Figure S16. Quantification of expression levels (RPKM) of Rian and Meg3, as well as Igf2 and Cdkn1c, representing imprinted genes misregulated in (a selection of) B6D2F1 EpiSC-NT lines.

Figure S17

Fig. S17a

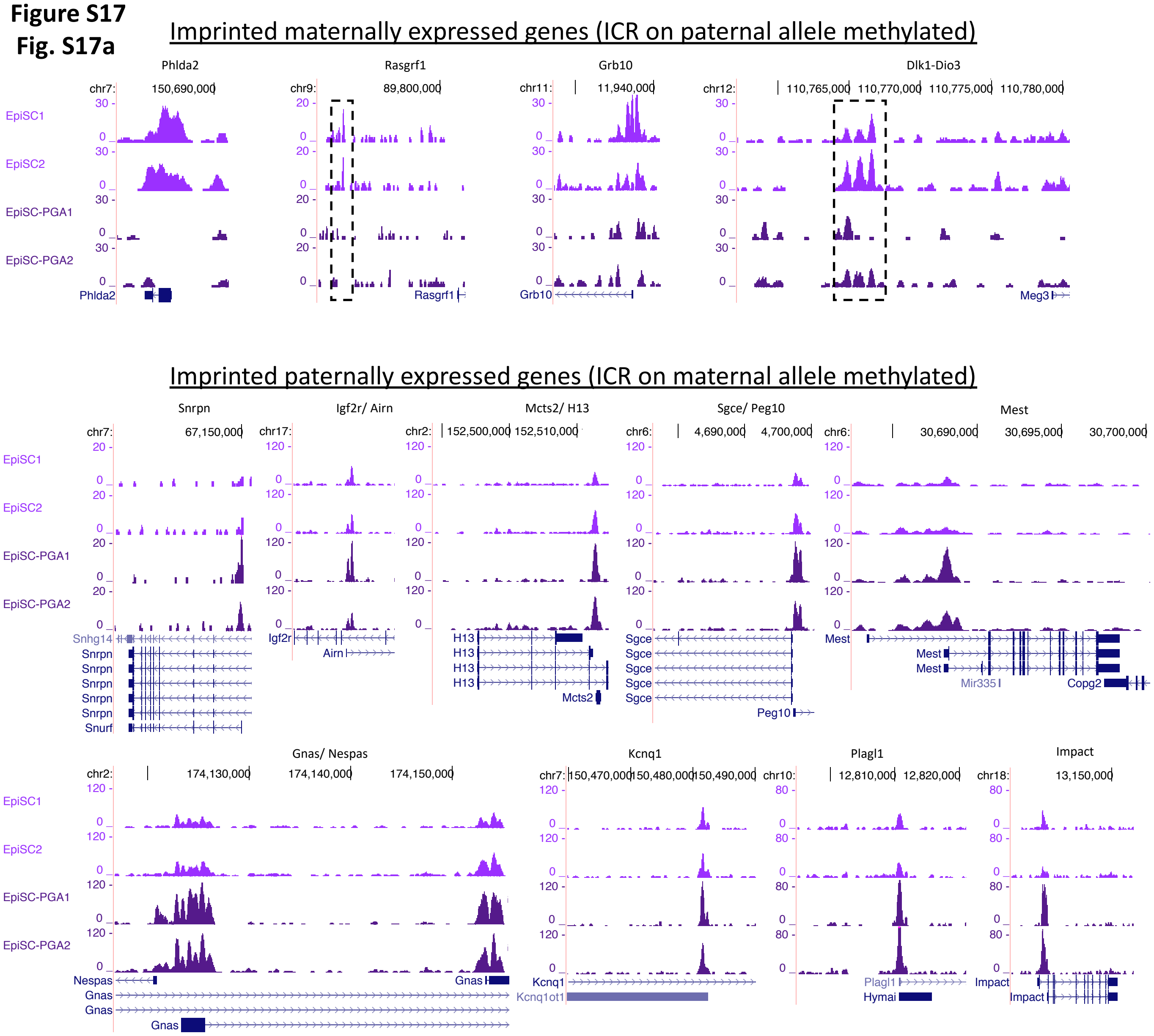

Fig. S17b

Imprinted maternally expressed genes (ICR on paternal allele methylated)								EpiSC-PGA/ EpiSC
Cluster	ICR genomic coordintates			EpiSC1	EpiSC2	EpiSC-PGA1	EpiSC-PGA2	
Phlda2	chr7	150686284	150689284	70	60	8	11	0.1
Rasgrf1	chr9	89773913	89774897	17	17	4	2	0.2
Grb10	chr11	11934713	11939586	139	72	54	35	0.4
Dlk1-Dio3	chr12	110763920	110767532	46	96	33	51	0.6

Imprinted paternally expressed genes (ICR on maternal allele methylated)								EpiSC-PGA/ EpiSC
Cluster	ICR genomic coordintates			EpiSC1	EpiSC2	EpiSC-PGA1	EpiSC-PGA2	
Snrpn	chr7	67148947	67150770	9	15	40	25	2.7
Igf2r/ Airn	chr17	12933454	12936613	95	96	206	101	1.6
Mcts2/ H13	chr2	152511127	152513884	67	118	201	211	2.2
Sgce/ Peg10	chr6	4695941	4699653	91	152	318	227	2.2
Mest	chr6	30684777	30690401	75	115	227	223	2.4
Gnas/ Nespas	chr2	174119610	174128127	207	288	770	655	2.9
Kcnq1	chr7	150480464	150483252	115	141	216	166	1.5
Plagl1	chr10	12807615	12812079	76	92	185	216	2.4
Impact	chr18	13129965	13133879	90	60	255	240	3.3

Figure S17. DNA methylation analysis of Imprinted Control Regions (ICRs). DNA methylation genome-wide profiles were generated using MethylCap-Seq. The four profiles were normalized to a total amount of 7,139,891 sequence tags to allow quantitative comparisons in the analysis performed for this figure. Coordinates of ICRs were obtained from Mikkelsen et al. [98] and Ferguson-Smith [99].

(a) DNA methylation over known ICRs in a genome browser view. For clarity, some of the ICRs are boxed. For the imprinted maternally expressed genes (ICR on paternal allele methylated), both PGA lines show a loss of DNA methylation as compared to the fertilized EpiSCs as expected for PGA lines. For the imprinted paternally expressed genes (ICR on maternal allele methylated), both PGA lines show ~2 fold increase in DNA methylation as compared to the fertilized EpiSCs as expected for PGA lines. Notably, for the ICRs for which there is one or more polymorphic site(s) to discriminate alleles, both EpiSC1 and EpiSC2 show the anticipated, monoallelic presence of DNA methylation on the expected allele (for Rasgrf1 (paternal) and Snrpn, Mcts2/ H13, Sgce/ Peg10, Plagl1 and Impact (all maternal) (data not shown)).

(b) Quantification (on tag counts) of peaks as shown in panel (a), including the fold change of the peak in the EpiSC-PGAs as compared to the fertilized EpiSCs (yellow header; green indicates decrease of peak, red indicates increase of peak)

Figure S18

Fig. S18a

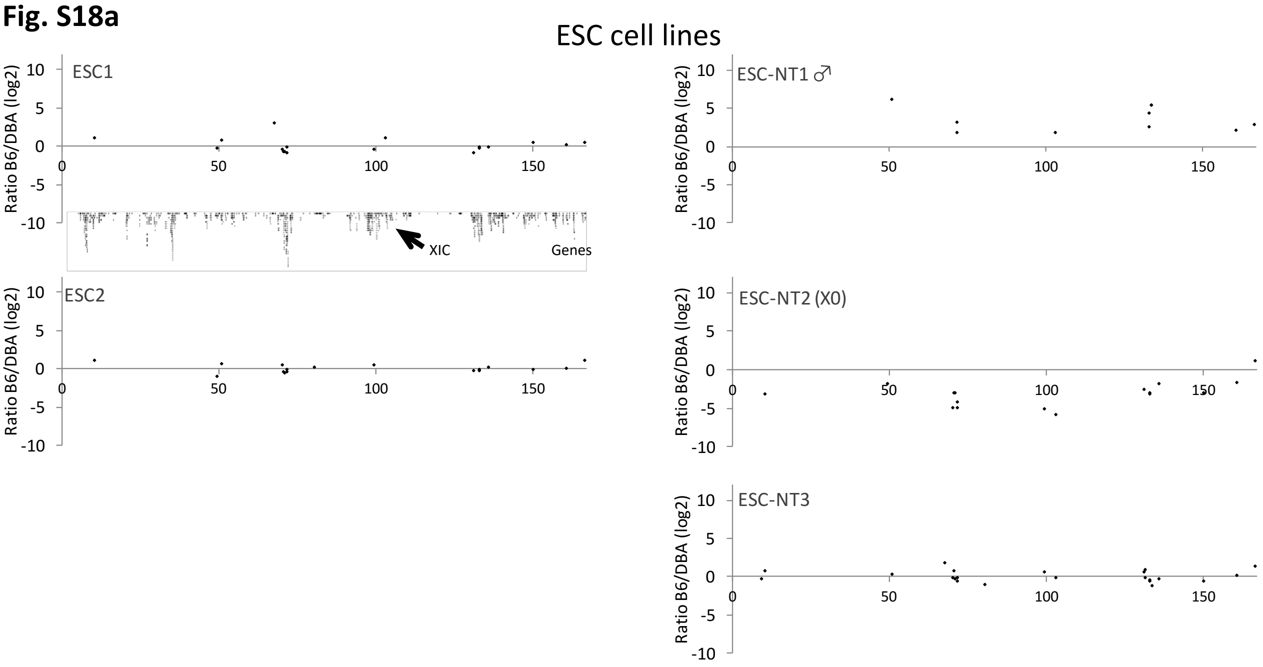

Fig. S18b

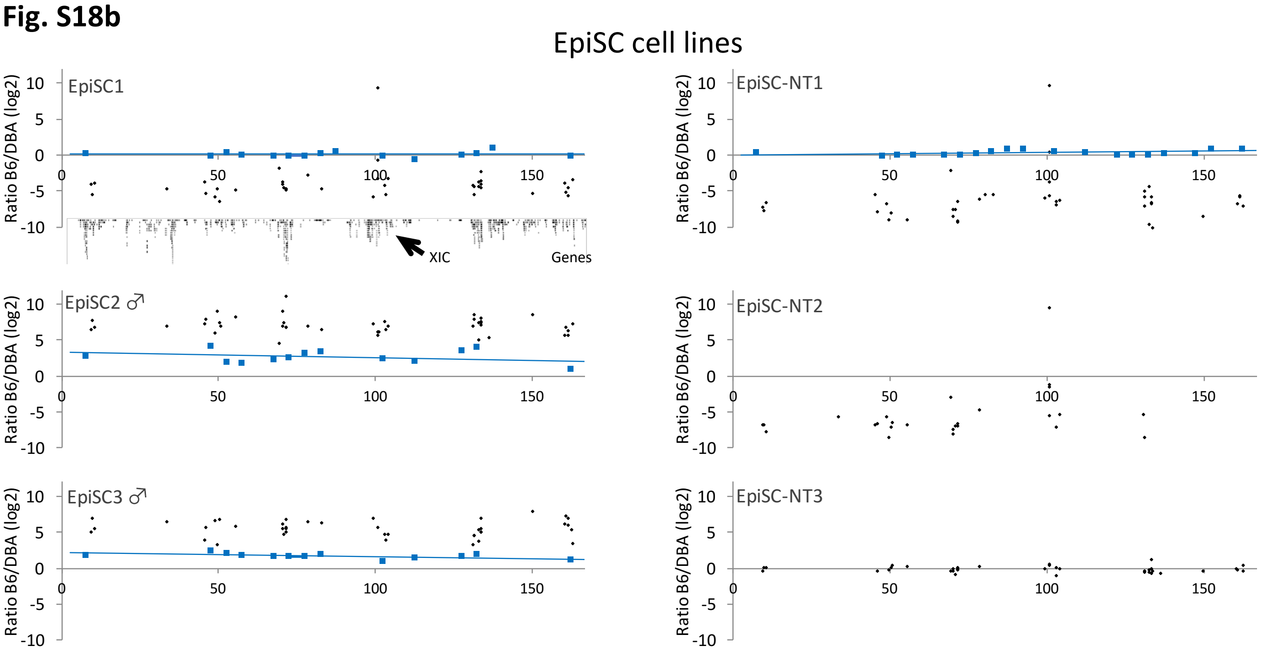

Figure S18. B6/DBA2 ratio per gene over the linear X chromosome (the X-axis representing genomic coordinates in MB) in ESCs **(a)** or EpiSCs **(b)**, similar to Fig. 4d. Each dot represents a gene. In blue the B6/DBA2 ratio obtained by DNA sequencing at 5MB resolution, confirming the presence of a B6 and DBA chromosome X in the female EpiSC1 and EpiSC-NT1 lines and the presence of a single B6 X chromosome in the male EpiSC2 and EpiSC3 lines. XIC = X inactivation center.
