# Supplementary material for: Allele-specific RNA-seq expression profiling of imprinted genes in mouse isogenic pluripotent states
Source: Epigenetics Chromatin. 2019 Feb 15;12:14. doi: 10.1186/s13072-019-0259-8 (PMC6376749; doi:10.1186/s13072-019-0259-8)
Supplement: Supplementary file 3 — Additional file 3: Table S2. Genotype and breakpoints of the B6D2F1 ESC-PGA lines used in this study as determined by RNA-Seq at 1 MB resolution. Red represents B6 on both alleles, blue represent heterozygous B6D2F1, yellow represents DBA2 on both alleles. A shift in genotype, indicated with a “,”, represents a recombination breakpoint that occurred in the B6D2F1 oocyte. [file 13072_2019_259_MOESM3_ESM.docx]

**Table S2 Dirks et al.:** Genotype and breakpoints of the ESC-PGA lines used in this study as determined by RNA-Seq at 1 MB resolution. Red represents B6 on both alleles, blue represent heterozygous B6D2F1, yellow represents DBA2 on both alleles. A shift in genotype, indicated with a “,”, represents a recombination breakpoint that occurred in the B6D2F1 oocyte.

|  | Breakpoints ESC-PGA1 | Breakpoints ESC-PGA2 | Breakpoints ESC-PGA3 |
| --- | --- | --- | --- |
| chr1 | 0-148,148-198 | 0-21,21-198 | 0-31,31-198 |
| chr2 | 24-182 | 0-69,69-182 | 5-182 |
| chr3 | 0-55,55-158 | 21-158 | 0-97,97-158 |
| chr4 | 0-137,137-156 | 0-15,15-156 | 0-72,72-156 |
| chr5 | 0-102,102-149 | 0-102,102-149 | 12-149 |
| chr6 | 0-53,53-150 | 47-118,118-150 | 0-101,101-150 |
| chr7 | 0-37,37-111,111-153 | 0-58,58-153 | 0-76,76-153 |
| chr8 | 0-40,40-132 | 31-132 | 14-132 |
| chr9 | 31-107,107-123 | 31-123 | 31-107,107-123 |
| chr10 | 0-8,8-128 | 0-30,30-117,117-128 | 0-45,45-128 |
| chr11 | 11-107,107-121 | 24-121 | 14-114,114-121 |
| chr12 | 0-36,36-121 | 0-54,54-120 | 14-121 |
| chr13 | 0-104,104-121 | 0-70,70-121 | 0-94,94-121 |
| chr14 | 0-20,20-122 | 0-100,100-122 | 0-42,42-122 |
| chr15 | 0-77,77-104 | 0-77,77-104 | 0-84,84-104 |
| chr16 | 0-23,23-99 | 0-60,60-99 | 0-28,28-99 |
| chr17 | 0-88,88-94 | 0-56,56-94 | 0-51,51-96 |
| chr18 | 0-53,53-87 | 30-87 | 0-48,48-87 |
| chr19 | 0-31,31-62 | 0-26,26-62 | 0-58,58-62 |
| chrX | 160-167 | 0-127,127-167 | 113-167 |
