# Supplementary material for: Allele-specific RNA-seq expression profiling of imprinted genes in mouse isogenic pluripotent states
Source: Epigenetics Chromatin. 2019 Feb 15;12:14. doi: 10.1186/s13072-019-0259-8 (PMC6376749; doi:10.1186/s13072-019-0259-8)
Supplement: Supplementary file 4 — Additional file 4: Table S3. Genotype and breakpoints of the B6D2F1 EpiSC-PGA lines as determined by genomic sequencing or RNA-Seq at 1 MB resolution. Red represents B6 on both alleles, blue represent heterozygous B6D2F1, yellow represents DBA2 on both alleles. A shift in genotype, indicated with a “,”, represents a recombination breakpoint that occurred in the B6D2F1 oocyte. The RNA-Seq genotyping algorithm identifies the correct position of the genomic breakpoints in 47% (9 of 19) and 64% (14 of 22) of the cases in EpiSC-PGA1 and EpiSC-PGA2, respectively, with an average accuracy of 1.3MB (EpiSC-PGA1) and 4.6MB (EpiSC-PGA2). The lower accuracy in EpiSC-PGA2 is mainly because three of the breakpoints are present in gene-poor loci where the RNA-Seq-based genotyping method lacks resolution. The distribution of relative expression from the B6 versus the DBA2 allele of the genes present within genomic regions genotyped as either homozygous B6, heterozygous B6/DBA2 or homozygous DBA2 of both EpiSC-PGA lines is present in Additional file 2: Fig. S7. [file 13072_2019_259_MOESM4_ESM.docx]

**Table S3 Dirks et al.:** Genotype and breakpoints of the B6D2F1 EpiSC-PGA lines as determined by genomic sequencing or RNA-Seq at 1 MB resolution. Red represents B6 on both alleles, blue represent heterozygous B6D2F1, yellow represents DBA2 on both alleles. A shift in genotype, indicated with a “,”, represents a recombination breakpoint that occurred in the B6D2F1 oocyte. The RNA-Seq genotyping algorithm identifies the correct position of the genomic breakpoints in 47% (9 of 19) and 64% (14 of 22) of the cases in EpiSC-PGA1 and EpiSC-PGA2, respectively, with an average accuracy of 1.3MB (EpiSC-PGA1) and 4.6MB (EpiSC-PGA2). The lower accuracy in EpiSC-PGA2 is mainly because three of the breakpoints are present in gene-poor loci where the RNA-Seq-based genotyping method lacks resolution. The distribution of relative expression from the B6 versus the DBA2 allele of the genes present within genomic regions genotyped as either homozygous B6, heterozygous B6/DBA2 or homozygous DBA2 of both EpiSC-PGA lines is present in Additional file 2: Fig S7.

|  | Genomic sequencing EpiSC-PGA1 | RNA-Seq EpiSC-PGA1 |  | Genomic sequencing EpiSC-PGA2 | RNA-Seq EpiSC-PGA2 |
| --- | --- | --- | --- | --- | --- |
| chr1 | 0-14,14-198 | 0-14,14-196 |  | 0-32,32-109,109-198 | 0-32,32-133,133-196 |
| chr2 | 17-101,101-172,172-182 | 30-101,101-171,171-182 |  | 0-59,59-145,145-182 | 0-59,59-145,145-182 |
| chr3 | 0-18,18-152,152-159 | 0-17, 17-158 (ND^*1^) |  | 0-37,37-159 | 0-37,37-158 |
| chr4 | 0-99,99-156 | 0-99,99-156 |  | 0-50,50-120,120-156 | 0-50,50-120,120-156 |
| chr5 | 0-91,91-151 | 0-90,90-149 |  | 0-47,47-140,140-149 | 0-47,47-140,140-149 |
| chr6 | 0-122,122-150 | 0-123,123-150 |  | 0-11,11-150 | 10-150 (ND^*2^) |
| chr7 | 0-78,78-153 | 0-74,74-153 |  | 0-38,38-153 | 0-37,37-153 |
| chr8 | 0-29,29-132 | 0-29,29-132 |  | 0-120,120-132 | 0-120,120-132 |
| chr9 | 13-125 | 31-123 |  | 31-53,53-117,117-125 | 31-53,53-117,117-123 |
| chr10 | 0-105,105-130 | 0-103,103-128 |  | 0-41,41-130 | 0-81,81-128 |
| chr11 | 0-64,64-122 | 0-67,67-121 |  | 0-37,37-114,114-121 | 0-36,36-114,114-121 |
| chr12 | 0-66,66-122 | 0-65,65-121 |  | 0-49,49-122 | 0-54,54-121 |
| chr13 | 0-35,35-121 | 0-33,33-121 |  | 16-121 (ND^*3^) | 0-15,15-121 |
| chr14 | 0-59,59-122 | 0-59,59-122 |  | 0-101,101-122 | 0-88,88-122 |
| chr15 | 0-84,84-104 | 0-84,84-104 |  | 18-104 | 23-104 |
| chr16 | 0-49,49-99 | 0-40,40-99 |  | 0-79,79-99 | 0-79,79-99 |
| chr17 | 0-44,44-96 | 0-44,44-96 |  | 0-40,40-96 | 0-38,38-96 |
| chr18 | 0-54,54-87 | 0-54,54-86 |  | 0-63,63-87 | 0-59,59-86 |
| chr19 | 0-11,11-62 | 0-11,11-62 |  | 0-31,31-62 | 0-31,31-62 |
| chrX | 45-167 | 0-167 |  | 45-167 | 0-126,126-167 |

*ND (Not Detected) due to very distal (*1) or proximal (*2), gene-poor chromosomal location with little coverage in RNA-Seq (*1 and *2) or genomic sequencing (*3)
